# In Vitro and In Silico Evaluation of the Potential Anti-Prostate Cancer Activity of *Rosmarinus officinalis* L. Leaf Extracts

**DOI:** 10.3390/ijms26104650

**Published:** 2025-05-13

**Authors:** Samantha Franchette B. Austria, Mon-Juan Lee, Kathlia A. De Castro-Cruz, Pang-Hung Hsu, Cheng-Yang Hsieh, Steven Kuan-Hua Huang, Po-Wei Tsai

**Affiliations:** 1 School of Chemical, Biological, and Materials Engineering and Sciences, Mapúa University, Intramuros, Manila 1002, Philippines; sfbaustria@mymail.mapua.edu.ph (S.F.B.A.); kadecastro@mapua.edu.ph (K.A.D.C.-C.); 2 Department of Chemical and Materials Engineering, National Kaohsiung University of Science and Technology, Kaohsiung 807, Taiwan; mjlee@nkust.edu.tw; 3 Department of Bioscience and Biotechnology, National Taiwan Ocean University, Keelung 202, Taiwan; phsu@ntou.edu.tw; 4 Department of Chemical and Materials Engineering, National I-Lan University, Yilan 260, Taiwan; d339108001@tmu.edu.tw; 5 Department of Food Science, National Taiwan Ocean University, Keelung 202, Taiwan; 6 Department of Medical Science Industries, College of Health Sciences, Chang Jung Christian University, Tainan 711, Taiwan; 7 Division of Urology, Department of Surgery, Chi Mei Medical Center, Tainan 710, Taiwan; 8 School of Medicine, College of Medicine, Kaohsiung Medical University, Kaohsiung 807, Taiwan

**Keywords:** DU-145, rosemary leaves, network pharmacology, molecular dynamics simulation, rosmarinic acid

## Abstract

Prostate cancer is one of the most prevalent cancer types diagnosed in older men. Investigations into traditional medicines like *Rosmarinus officinalis* L., popularly known as rosemary, are a current research interest due to its anti-cancer properties. This study investigates the cytotoxicity of aqueous and ethanolic rosemary leaf extracts in DU-145 cells and the interaction of its active metabolites with key prostate cancer targets using an in silico approach. The water extract of rosemary leaves showed greater cytotoxicity than the ethanol extract, with IC_50_ values of 1.4140 ± 0.1138 mg/mL and 1.8666 ± 0.0367 mg/mL, respectively; the highest cytotoxic effects for both extracts were observed at 5 mg/mL. These findings indicate significant cytotoxic differences based on concentration and solvent. Network pharmacology identified 37 genes linked to prostate adenocarcinoma, highlighting key genes like *EGFR*, *TP53*, *ERBB2*, *IGFBP3*, *MMP-2*, *MMP-9*, *HDAC6*, *PDGFRB*, and *FGFR1*. Molecular dynamics simulations and binding energy calculations revealed strong interactions between carnosol and rosmarinic acid with these targets, with TP53–carnosol showing the most stable conformation. Rosmarinic acid was identified as a promising candidate due to its low toxicity. This study demonstrates the potential anti-prostate cancer properties of rosemary leaf extracts for further investigations on the development of drugs against prostate cancer.

## 1. Introduction

Cancer is a significant cause of death globally, accounting for around 10 million deaths in the year 2020, as reported by the World Health Organization (WHO) [1]. Numerous studies demonstrate that males are at higher risk than women to be diagnosed and die from cancer, with prostate-related cancer being the most prevalent form in men [2,3]. Men over 40 frequently suffer from multiple pathologies affecting their prostates, including benign prostatic hyperplasia (BPH), which is commonly characterized by an enlarged prostate and may be a precursor to cancer. Prostate cancer (PCa) is a disease that primarily affects older men worldwide, and its incidence has risen in recent years, affecting over 1.28 million men, leading to 358,989 deaths in the year 2018 [4,5]. It has been suggested that risk factors of PCa include age, ethnicity, family history, carrying a pathogenic germline variation in a PCa predisposition gene, and obesity [6,7,8]. Many studies have assessed the risk of PCa based on symptoms and indications since, as with other malignancies, early detection is crucial for proper clinical treatment, prevention of mortality, and reduction of morbidity rates of PCa [9].

Due to significant developments in science and technology, different treatment approaches have been established for PCa treatment, including surgery, chemotherapy, radiotherapy, local therapy, and endocrine therapy [10,11]. The development of novel therapeutics, including second-generation antiandrogens, bone-modifying agents (BMAs), and poly(ADP-ribose) polymerase (PARP) inhibitors, have significantly enhanced the state of modern treatment for advanced PCa alongside increased patient survival [12,13,14,15]. Substantially, traditional treatment for PCa has been widely delved into in various studies in recent years, as traditional plants are known to have been widely used to cure diseases, which led to the increase in drug discovery and studies using plant extracts [16]. Plants are potential sources of natural bioactive compounds and secondary metabolites essential for human health due to their biological effects, including antioxidant, anti-inflammatory, and anticarcinogenic activities [17,18]. With this, several studies evaluate and assess the development of chemotherapeutic agents from herbal medicines using plant extracts against PCa with in vitro cell-based assays using PCa cell lines such as PC-3 and DU145 [19,20,21].

Plants in the Lamiaceae family, which includes several herb and shrub genera, are currently the subject of extensive research due to their notable anticancer activity. It was recently argued that their extracts may have antiproliferative potential toward PCa cells in vitro [16,22,23]. One of these is *Rosmarinus officinalis* L., commonly known as rosemary. Rosemary is a versatile plant that can be grown in various climates and soil conditions and is considered an ancient herb widely cultivated for its medicinal purposes [24,25]. Previous research has shown the cancer-fighting potency of rosemary extracts involves several mechanisms, including immune response regulation, antioxidant activity, and anti-inflammatory responses, using several purification and extraction approaches, which consequently revealed that rosemary is packed with bioactive phenolic compounds such as carnosic acid, ursolic acid, carnosol, and rosmarinic acid [26,27,28]. Due to its ability to interfere with hormone receptors, particularly the androgen receptor (AR), a target for prostate tumors, which are classified as either AR positive or negative, rosemary has a noteworthy relation with PCa [29]. Limited data have been established to evaluate the effects of rosemary extract on AR-negative PCa cells despite having been reported that earlier studies showed that it could reduce cell survival, proliferation, and viability and increase apoptosis in various cancer cell lines [30].

Network pharmacology and molecular docking, which seek to comprehend drug actions and interactions with multiple targets, have become increasingly helpful drug discovery techniques as technology advances. The “one-target, one-drug” paradigm has been replaced with the more practical “multi-target drug” approach, which is in line with the concepts and methods of traditional Chinese medicine (TCM) [31,32]. These techniques facilitate the construction of intricate polypharmacology networks based on lead compounds, biological processes, and bioactive target proteins by assisting in identifying potential targets and understanding the mechanisms that govern the effectiveness of bioactive compounds against cancer [33,34].

Given the findings regarding the anticancer potential of rosemary in the existing literature, this study focused on evaluating the potency of rosemary against PCa. The cytotoxic effects of rosemary water and ethanol extracts using an in vitro cell-based assay in the androgen-independent PCa cell line DU145 are investigated in this paper. Furthermore, the effectiveness of its metabolite in targeting several proteins promoting PCa progression is examined using a network pharmacology and molecular docking approach. This paper explores the use of rosemary in the medicine and pharmaceutical field, particularly in the context of drug discovery and development for PCa.

## 2. Results

### 2.1. Identification of Major Components of Rosemary

**Figure 1:**
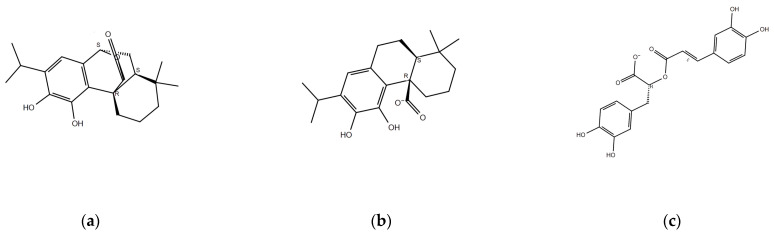
Chemical structures of selected bioactive compounds found in *Rosmarinus officinalis* L. retrieved from Maestro: (**a**) carnosol, (**b**) carnosic acid, and (**c**) rosmarinic acid.

The major bioactive compounds present in *Rosmarinus officinalis* L. selected for this study are carnosol (COH), carnosic acid (CA), and rosmarinic acid (RA). These phytochemicals have been widely reported due to their antioxidant, anti-inflammatory, and anticancer activities, particularly their effects on PCa-related pathways. These compounds have demonstrated the ability to inhibit tumor growth and modulate key molecular targets involved in cancer progression. Figure 1 illustrates the chemical structures of COH (Figure 1a), CA (Figure 1b), and RA (Figure 1c).

**Figure 2:**
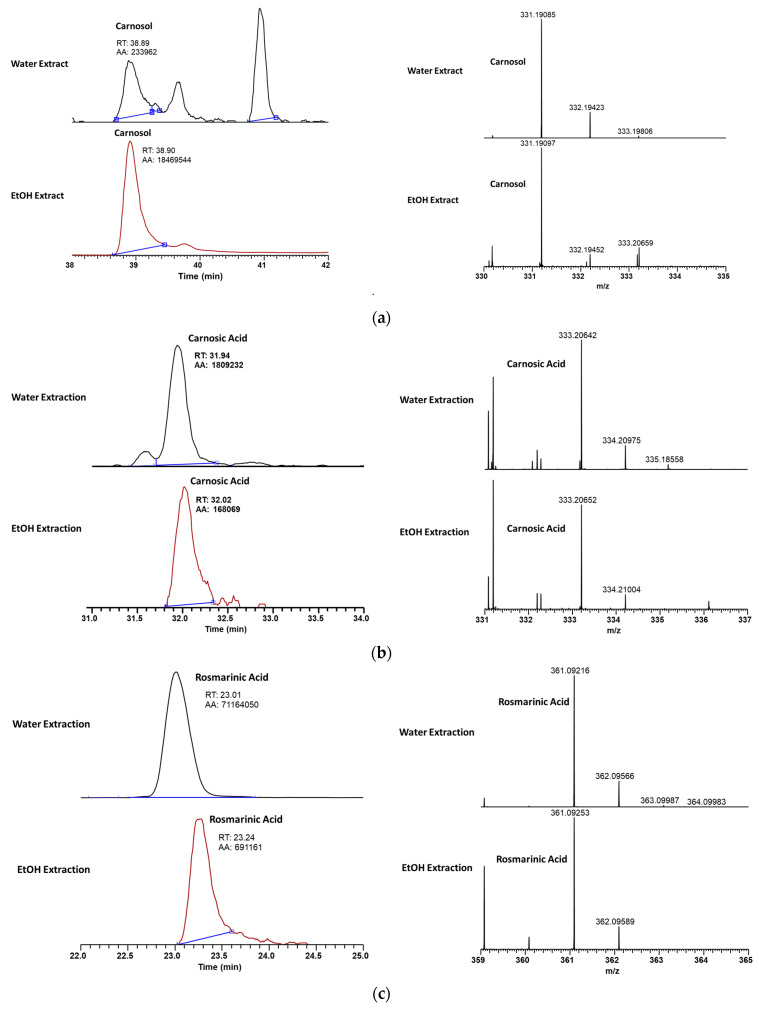
Comparative LC-MS analysis of carnosol (**a**), carnosic acid (**b**), and rosmarinic acid (**c**) in water and ethanol extracts of rosemary leaves.

Figure 2 shows the comparative LC-MS analysis of COH (Figure 2a), CA (Figure 2b), and RA (Figure 2c) in water and ethanol extracts of rosemary leaves. The results indicate that water extraction yields significantly higher amounts of RA (RT = 23.01 min, AA = 71,164,050) compared to ethanol extraction (RT = 23.24 min, AA = 691,161). Mass spectrometry confirms the presence of RA in both extracts, with a molecular ion peak at m/z 361.09216. Despite slight differences in retention time, both methods successfully isolate RA, but water extraction proves more efficient in obtaining higher concentrations.

### 2.2. Anti-Prostate Cancer Activity of Rosemary Extracts

**Figure 3:**
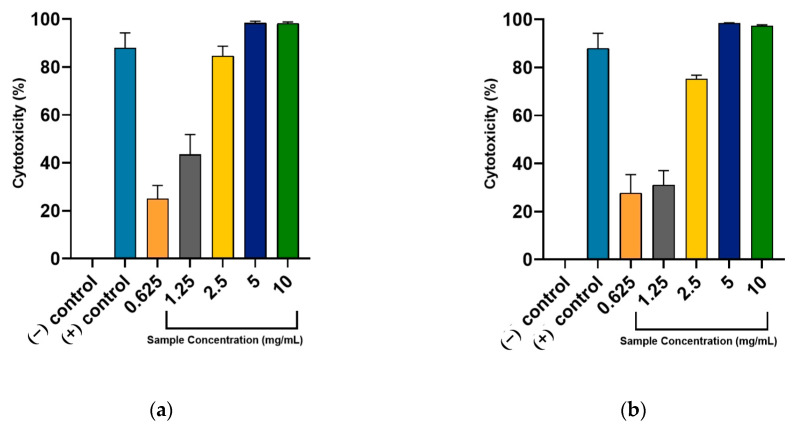
Concentration-dependent cytotoxicity of rosemary leaf extracts on DU-145 after a 24-h treatment period: (**a**) water extract (RL-W) and (**b**) ethanol extract (RL-E). The positive (+) control is 1 mg/mL 5-FU, while the negative (−) control includes only the media. These results are expressed as mean ± standard deviation. Sample concentration and solvent interaction were significantly different based on the two-way ANOVA analysis. Additionally, it was revealed that treatment with varying sample concentrations and solvent extracts causes significant sources of variation in the resulting cytotoxic effects of the plant extract.

The findings suggest a dose-dependent response in DU-145 cells across varying concentrations. Higher doses of both water and ethanol extracts induced cytotoxic effects that significantly differed from the negative control, with the water extract showing the most pronounced effect. As illustrated in Figure 3a,b, concentrations of 5 and 10 mg/mL of both extracts surpassed the anti-PCa activity of the standard drug 5-fluorouracil (5-FU). Their cytotoxic effects were almost equal, but a higher effect was observed when 5 mg/mL of sample was used for treatment. Substantially, a higher half-maximal cytotoxic concentration expressed as IC_50_ was observed for RL-W (1.4140 ± 0.1138 mg/mL) compared with RL-E (1.8666 ± 0.0367 mg/mL). Thus, water extract was a more potent treatment option against DU-145 cells than the ethanol extract.

### 2.3. Network Pharmacology

#### 2.3.1. Protein–Protein Interaction Network of Identified Gene Targets

**Figure 4:**
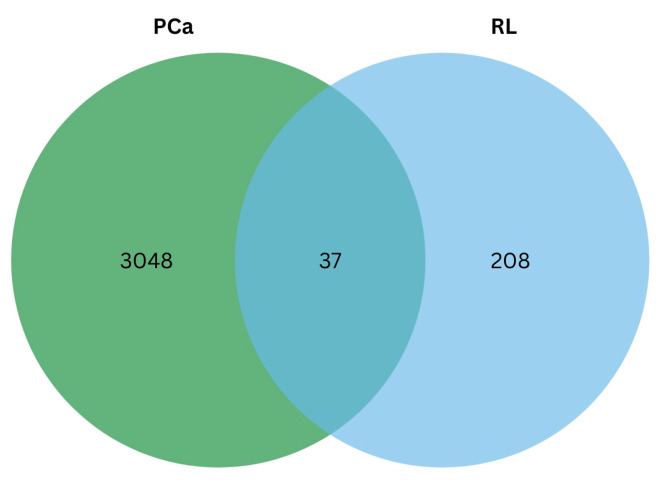
Overlapping gene set between predicted targets of *Rosmarinus officinalis* L. metabolites (blue) and prostate cancer (PCa) genes (green).

Primary active metabolites (COH, CA, and RA) found in rosemary leaf extracts using water and ethanol solvent systems were obtained from the existing literature [29,35,36,37,38,39,40]. The over-expressed and under-expressed prostate adenocarcinoma (*PRAD*) genes and differentially expressed genes (*GSE179324*) versus normal prostate cells for the DU-145 cell line were identified. Figure 4 shows that the total number of shared genes between significant rosemary metabolites in leaf extracts and PCa target genes is 37.

**Figure 5:**
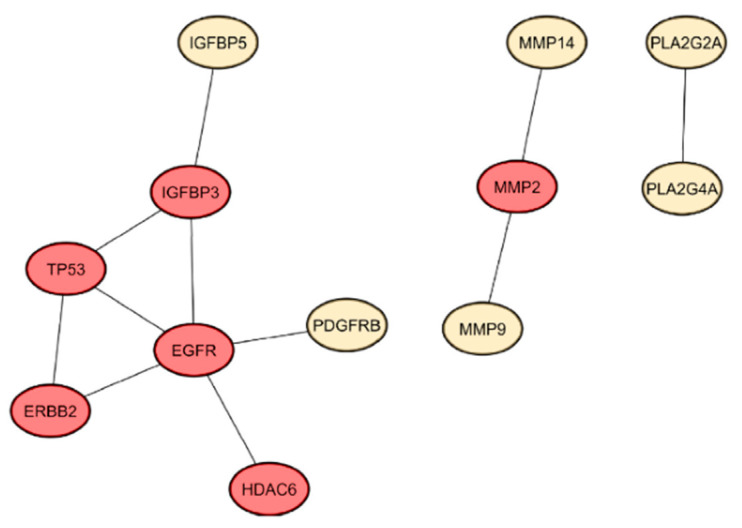
PPI network of intersected genes where red-colored nodes are the selected protein targets obtained from centrality analysis.

A protein–protein interaction (PPI) network was constructed in Figure S1 using these overlapping genes with an interaction score of 0.4 in STRING. An increase in the minimum required interaction score of 0.9 (highest confidence) gave network statistics reporting a PPI enrichment p-value of 3.88 × 10^−4^ with 28 connected components. This result suggests that the target proteins interact more with themselves than expected. Centrality analysis and evaluation were performed considering BC, CC, MCC, DMNC, and DC metrics to identify essential proteins for use as key targets to evaluate the potency of selected RL metabolites against PCa. Figure 5 exhibits a constructed PPI network with the top six proteins resulting from the analysis with an interaction score of 0.9. The target genes were epidermal growth factor receptor (*EGFR*), tumor protein p53 (*TP53*), insulin-like growth factor-binding protein 3 (*IGFBP3*), receptor tyrosine-protein kinase erbB-2 (ERBB2), matrix metalloproteinase-2 (*MMP-2*), and histone deacetylase 6 (*HDAC6*).

#### 2.3.2. Enrichment Analysis

**Figure 6:**
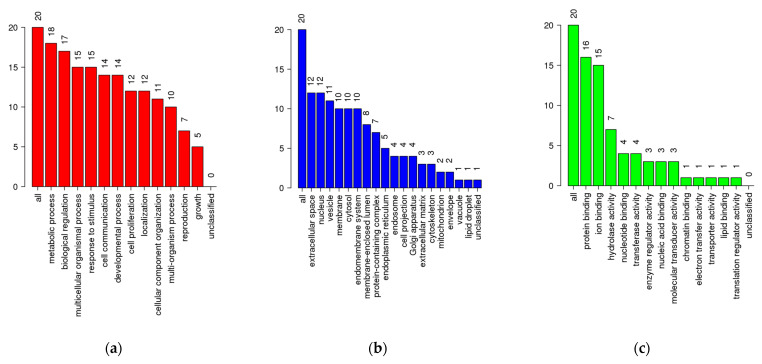
Gene Ontology slim summaries for (**a**) BP, (**b**) CC, and (**c**) MF.

The 37 intersected predicted target genes obtained from the network pharmacology analysis were further assessed using enrichment analysis. Gene Ontology (GO) slim summary enrichment analysis results from WebGestalt are displayed in Figure 6, comprising three categories: Biological Process (BP), Cellular Component (CC), and Molecular Function (MF) [41]. In the BP category (Figure 6a, Table S3), most genes were associated with biological regulation, metabolic processes, response to stimulus, and cell communication. These findings suggest that the predicted targets of the bioactive compounds found in rosemary are integral to regulating cell proliferation, apoptosis, and core signal transduction mechanisms underlying PCa development and progression. Notably, enrichment in cell proliferation and development processes further highlights the potential of rosemary to influence cancer cell growth and differentiation.

Within the CC (Figure 6b, Table S4) and MF (Figure 6c, Table S5) categories, a substantial portion of genes were linked to membrane components, extracellular space, and protein-containing complexes, as well as protein binding, hydrolase activity, and nucleic acid binding. These results underscore the importance of membrane-bound receptors and protein–protein interactions in mediating the anti-cancer effects of rosemary. By targeting genes involved in membrane signaling and complex formation. Rosemary compounds may disrupt critical pathways that support PCa cell survival, metastasis, and adaptation to the tumor microenvironment. The GO enrichment analysis provides mechanistic insight into how rosemary may exert its anti-PCa effects, supporting its potential as a multi-target agent that modulates essential biological pathways in prostate cancer.

**Figure 7:**
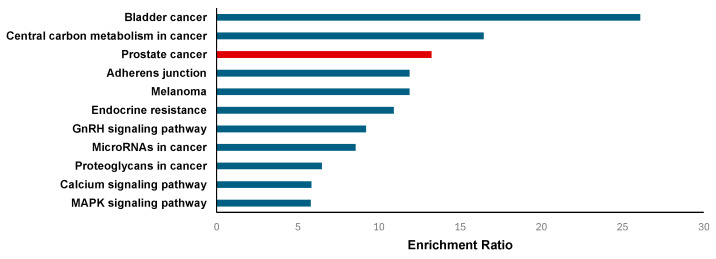
Bar chart of the top enriched terms for the KEGG pathway based on the enrichment ratio. The red-colored indicates the enrichment ratio of PCa.

Furthermore, the Kyoto Encyclopedia of Genes and Genomes (KEGG) pathway enrichment analysis of the 37 intersected genes identified 11 significantly enriched pathways (Figure 7), with the PCa pathway (Figure S2) prominently featured in red color. This pathway included six crucial genes—*EGRF*, *ERBB2*, *TP53*, fibroblast growth factor receptor 1 (*FGFR1*), matrix metallopeptidase 9 (*MMP-9*), platelet-derived growth factor receptor beta (*PDGFRB*)—all of which are known players in prostate tumorigenesis. *EGFR*, *ERBB2*, and FGFR1 are known to promote tumor growth and survival independently of AR signaling. This is highly relevant for AR-negative PCa, which lacks responsiveness to androgen deprivation therapies and often relies on alternative growth and survival pathways for progression. These genes regulate critical processes such as cell proliferation, apoptosis, extracellular matrix remodeling, and angiogenesis, suggesting that bioactive compounds of rosemary may exert anti-cancer effects by modulating these oncogenic pathways [42]. Other enriched KEGG pathways (Figures S3 and S4) include bladder cancer, central carbon metabolism in cancer, MAPK signaling pathway, calcium signaling pathway, GnRH signaling pathway, and pathways related to cancer resistance and microRNAs. The enrichment of metabolic pathways and signaling cascades such as MAPK and calcium signaling highlights the multifaceted mechanisms through which rosemary may influence prostate cancer progression, including altering cellular metabolism, signal transduction, and hormonal regulation [43].

Complementary Reactome pathway (Table S7, Figure S7) analysis further supported these findings by identifying pathways with high enrichment ratios related to triglyceride catabolism, activation of matrix metalloproteinases, triglyceride metabolism, constitutive signaling by aberrant PI3K in cancer (Figure S6), and PI3K/AKT signaling in cancer pathways (Figure S7). The PI3K/AKT pathway is particularly notable for its role in promoting cell survival and proliferation in PCa, and its modulation by rosemary targets suggests a potential mechanism for inhibiting tumor growth [42]. Additionally, the activation of matrix metalloproteinases aligns with the involvement of *MMP9* in extracellular matrix remodeling and metastasis [44].

### 2.4. Molecular Docking Simulation and MM-GBSA Calculation

The docking validation for multiple PCa target proteins was performed by comparing the RMSD values of the docked poses to the experimentally established co-crystallized ligands (Table 1). In general, accurate docking predictions are indicated by RMSD values less than 2 Å. *EGFR* (PDB ID: 1M17) had an RMSD of 1.2451 Å, and *ERRB2* (PDB ID: 3PP0) had a great RMSD of 0.6354 Å. *HDAC6* (PDB ID: 5EDU) and *MMP-2* (PDB ID: 1HOV) had strong RMSDs of 0.7883 Å and 1.3172 Å, respectively. These results are highly favorable for the majority of targets. Additionally, *MMP-9* (PDB ID: 1GKC) and *FGFR1* (PDB ID: 7WCL) displayed reasonable RMSD values of 1.4014 Å and 1.7018 Å. PDGFRB (3MJG) demonstrated an acceptable RMSD of 1.3752 Å, whereas *TP53* (Y220C mutation, PDB ID: 2VUK) displayed a very good RMSD of 0.3593 Å. On the other hand, given that there was no co-crystallized ligand for *IGFBP3* (PDB ID: 7WRQ), docking validation using RMSD comparison was not an appropriate approach. Since the available PDB structure for this protein does not contain a co-crystallized ligand, the active site was identified using the SiteMap with the most negative binding energy from Maestro. Overall, the validation of the re-docking approach suggests that the method produces dependable and precise predictions for protein targets.

The MM-GBSA binding energy (in kcal/mol) for each protein–ligand complex is reported in Table 2, where the docking was followed by a subsequent calculation of the binding energy, which can be correlated to affinity. The binding energy values contribute valuable understanding regarding the strength of interactions between the ligand and their respective protein targets—a more negative value indicates a stronger binding [45]. COH demonstrates strong binding to several targets, including *HDAC6* (−49.41 kcal/mol), *TP53* (−41.87 kcal/mol), and *IGFBP3* (−42.85 kcal/mol), suggesting that it may be a promising candidate for these proteins. Additionally, COH demonstrates a competitive binding energy for *EGFR* (−35.04 kcal/mol) as compared to the other ligands, suggesting that it also has a considerable affinity for this target. In comparison, CA displays less favorable binding across most targets, particularly for *ERBB2* (4.69 kcal/mol), which suggests weak binding interactions. RA presents robust binding to several protein targets, including *ERBB2* (−46.00 kcal/mol), *MMP-9* (−42.28 kcal/mol), and IGFBP3c (−47.23 kcal/mol), highlighting its potential as a strong binder for these proteins. In contrast, 5-FU presents moderate binding energies between −20 and −31 kcal/mol, with its weakest binding observed when it is docked with *TP53* (−16.48 kcal/mol).

Significantly, the proteins in complexes with reference drugs exhibit the most negative binding energies, indicating strong affinity. These ligands interact strongly with their respective receptors. These findings provide an essential threshold for assessing docking accuracy and ligand potency considering that they are consistent with known high binding affinities of reference drugs. When compared to the co-crystallized ligands, RA and COH reveal favorable and competitive binding behaviors for certain targets as stated previously. Given that their binding energies are comparable to or stronger than those of the reference drugs, this report suggests that COH and RA are promising therapeutic candidates for further investigations, particularly for targets such as *HDAC6* and *TP53*.

**Figure 8:**
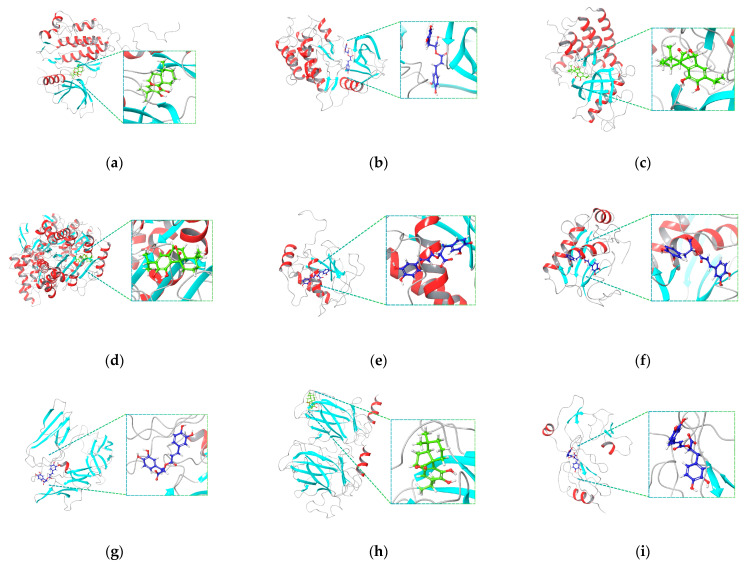
Representative protein–ligand complexes with ligands bound in the active site of nine target proteins. The protein is shown using a ribbon representation, and the ligand is displayed in the ball-and-stick model. For each protein, only the complex with the most negative binding energy for the respective ligand is presented. Insets provide a magnified view of the ligand within the binding site. Panels correspond to the following complexes: (**a**) *EGFR* (PDB ID: 1M17)–COH, (**b**) *ERBB2* (PDB ID:3PP0)–RA, (**c**) *FGFR1* (PDB ID: 7WCL)–COH, (**d**) *HDAC6* (PDB ID: 5EDU)–COH, (**e**) *MMP-2* (PDB ID: 1HOV)–RA, (**f**) *MMP-9* (PDB ID: 1GKC)–RA, (**g**) *PDGFRB* (PDB ID: 3MJG)–RA, (**h**) *TP53* (PDB ID: 2VUK)–COH, and (**i**) *IGFBP3* (PDB ID: 7WRQ).

Figure 8a–i depicts the ligand bound in the active site pocket of the protein, with the protein secondary structure shown as ribbons and the ligand displayed in the ball-and-stick model to highlight atomic interactions. Two-dimensional representations of the interactions of these receptor–ligand interactions are illustrated in Figure S8a–i. It was observed that the most frequent interactions are hydrogen bonds involving polar residues such as lysine, arginine, aspartate, asparagine, and glutamate interacting with phenolic or carboxyl groups of the ligand. Moreover, π–π stacking was common between ligand aromatic rings and aromatic residues such as tyrosine and phenylalanine, which significantly contributed to binding stability. The π–cation interactions mainly involved lysine reacting with aromatic rings of COH. On the other hand, the prominent interaction for complexes with RA as the ligand involves electrostatic interactions because of the acidic carboxylate groups interacting with glutamate residues. Hydrophobic contacts were also evident as these stabilize COH through interaction with the nonpolar residues valine and leucine.

### 2.5. Molecular Dynamics Simulation

For the MD simulations, three receptor–ligand complexes—*EGFR*–COH, ERBB2–RA, and *TP53*–COH—were selected. These receptors were identified in KEGG pathway analysis and ranked among the top proteins in the PPI network. The selection was based on their relevance to the biological pathway, prominence in the protein network, and calculated binding energy. The structural stability (RMSD, RMSF), ligand properties (rGyr, SASA, PSA, MOLSA), and receptor–ligand interactions were analyzed to assess the stability and dynamics of the complexes.

#### 2.5.1. *EGFR*–COH Complex

**Figure 9:**
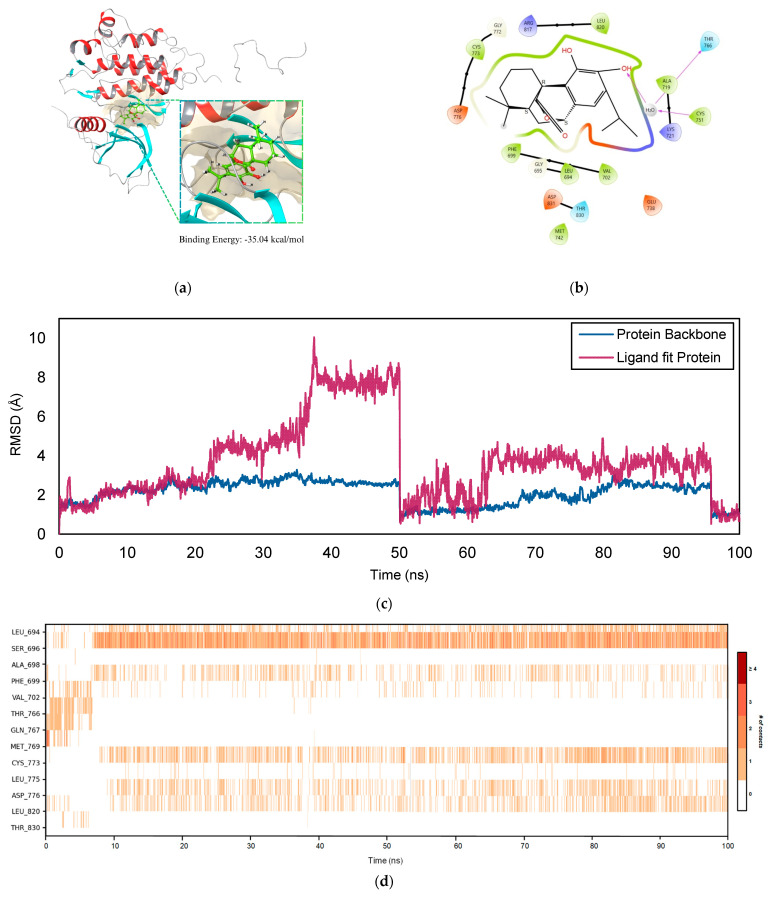
*EGFR*–COH complex. (**a**) Docking pose of the ligand within the protein binding site with the calculated MM-GBSA binding energy reported in kcal/mol. (**b**) Two-dimensional interaction diagram illustrating detailed protein–ligand contacts. The stereochemical configuration with chiral centers assigned as (S) and (R) and double bonds designated as (E) and (Z). (**c**) RMSD plot of the protein–ligand complex over a 100 ns MD simulation, indicating structural stability. (**d**) Chronological map of protein–ligand contacts over a 100 ns MD simulation, depicting the persistence and dynamics of specific residue interactions throughout the trajectory. Residue contact frequency is represented by color, with darker shades of orange on the scale to the right indicating more frequent interactions.

Figure 9a reveals the binding site surface of the *EGFR*–COH complex wherein −35.04 kcal/mol was reported. Moreover, Figure 9b highlights the data on the interaction between COH and EGFR, emphasizing the essential stabilizing forces involved. Hydroxyl groups on COH participate in hydrogen bonding, including one bond mediated by a water molecule interacting with residues such as THR 766. Additionally, hydrophobic interactions with residues like LEU 820, PHE 699, and MET 742 contribute to stabilizing the molecule within the binding pocket. The stability of the complex is further enhanced by π–π stacking interactions with PHE 699 and electrostatic interactions with charged residues, including ASP 776 and GLU 738. The high affinity of COH for *EGFR* is demonstrated by its fitting into the *EGFR* binding pocket, as well as through water-mediated interactions and main chain contacts with residues such as ALA 719 and LYS 721. These interactions suggest a potential inhibitory effect on receptor activity, which could have significant implications for therapeutic applications in cancer treatment.

The root mean square deviation (RMSD) for the *EGFR*–COH complex over 100 ns is illustrated in Figure 9c. The RMSD values indicate that the system undergoes significant conformational changes, particularly during the early stages of the simulation. At 10 ns, the RMSD for the protein backbone is relatively high at 7.929 Å, suggesting considerable structural flexibility as the protein adjusts to the presence of the ligand. Similarly, the ligand’s RMSD starts at 5.442 Å, reflecting the mobility within the binding site as it adapts its conformation to fit into the protein’s active pocket. Between 20 ns and 40 ns, the RMSD values for the protein and the ligand continue to rise. The protein reaches a peak RMSD of 12.423 Å at 30 ns, while the ligand fluctuates but remains around 7.86–8.461 Å. These values indicate that the system is undergoing significant adjustments, with the protein and the ligand exploring various conformations. The continued increase in RMSD suggests that the complex has not yet settled into a stable configuration, and both the protein and ligand are experiencing flexibility as they search for an energetically favorable arrangement.

However, the ligand and protein experience a sharp decrease in RMSD after 50 ns, indicating that the system is stabilizing. Notably, the protein’s RMSD drops significantly from 12.423 Å at 30 ns to 1.515 Å at 60 ns. This decline suggests that the protein backbone has adopted a more stable conformation, with minimal variations observed thereafter. Similarly, the ligand’s RMSD decreases from 7.86 Å to 2.442 Å during the same time frame, indicating that the ligand has reached a stable conformation within the binding pocket. Between 60 ns and 100 ns, the RMSD values for both the protein and ligand remain consistently low, ranging from 1.427 Å to 2.452 Å for the protein and from 1.26 Å to 2.78 Å for the ligand. These low and stable RMSD values demonstrate that the *EGFR*–COH complex has achieved structural stability, with both components exhibiting well-defined and stable conformations, indicating a strong protein–ligand interaction.

Figure 9d provides a detailed timeline representation of specific contacts between COH and *EGFR* over a period of 100 ns. During the initial period of 10 ns, contacts are made with residues VAL 702, THR 766, and GLN 767. Noticeably, as the interaction between the ligand and receptor stabilizes under 100 ns, evident residues, mainly including SER 696, CYS 773, and LEU 694, maintain persistent contacts. This suggests that these residues are key anchors in the stabilization of the ligand in the binding site. The close contact and proper orientation of the ligand to specific residues in the binding site were maintained through hydrogen bonding and water bridge interactions, contributing to the stability of the complex. This can be observed from the decreasing fluctuations in RMSD values as it reaches 100 ns.

Figure S9 presents the ligand RMSF for the *EGFR*–COH complex over 100 ns. Most atoms in the *EGFR*–COH complex exhibit comparatively little variability in their ligand RMSF values, ranging from 0.98 to 1.24 Å. This indicates that the ligand maintains a stable conformation while undergoing minor structural modifications during the simulation. The RMSF values for atoms 1 (1.0586 Å), 6 (1.0208 Å), and 12 (0.9876 Å) fall within the lower range, suggesting that these regions exhibit relative rigidity and maintain their position within the protein binding pocket. In contrast, atoms 7 (1.1478 Å) and 16 (1.2084 Å) show significantly greater RMSF values, indicating small but noticeable flexibility in these areas. This flexibility may allow specific regions of the ligand to undergo minor conformational changes that enhance interactions with the protein. Despite these slight variations, the overall stability of the ligand suggests that *EGFR* and COH form a stable, physiologically relevant complex with minimal structural flexibility.

Additionally, Figure S10 exhibits an analysis of the conformational dynamics of the ligand properties. During the 100 ns simulation, the radius of gyration (rGyr) remained relatively constant, fluctuating between 39.6 Å and 40.49 Å, which corresponds to a variation of approximately 2.24%. This stability suggests that the *EGFR*–COH complex did not undergo significant unfolding and maintained its compact structure. The peak rGyr value of 40.49 Å observed at 60 ns likely indicates a brief conformational change due to ligand movement or local flexibility. However, the rGyr readings consistently returned to baseline values of around 39.8–39.9 Å, demonstrating that the complex was well-structured.

The MolSA varied between 2956.92 Å^2^ and 3009.36 Å^2^, indicating a variation of approximately 1.8% and demonstrating minimal fluctuation. The lowest value of 2956.92 Å^2^, recorded at 70 ns, suggests a slight contraction at the protein–ligand interface. In contrast, the highest MolSA value of 3009.36 Å^2^, observed at 80 ns, indicates a slight outward movement of surface residues or an expansion induced by the ligand. These subtle variations contribute to the structural stability of the complex.

Comparatively, the SASA showed more variation (1031.39 Å^2^ to 1263.4 Å^2^, ~22% variation). At 60 ns, the lowest SASA value of 1031.39 Å^2^ suggests a brief shielding of hydrophilic regions, likely due to enhanced hydrophobic interactions with the ligand or rearrangements of surface residues. In contrast, the highest SASA value (1263.4 Å^2^ at 100 ns) suggests a temporary increase in solvent exposure, which dynamic variations in ligand location or surface residues could bring on. Despite this variance, there was no discernible destabilization since the SASA alterations stayed within the range of typical protein–ligand interactions.

The PSA stayed primarily constant, ranging from 1228.8 Å^2^ to 1294.51 Å^2^ (~5% fluctuation). The PSA value of 1294.51 Å^2^ at 30 ns is the highest, indicating increased exposure to polar residues, which may enhance hydrogen bonding with the ligand or solvent. Conversely, the lowest value, 1228.8 Å^2^ at 20 ns, most likely reflects a brief decrease in polar accessibility brought on by transitory side-chain rearrangements. Given these minimal variations, the overall stability of PSA highlights the preservation of crucial polar interactions necessary for complex stability.

#### 2.5.2. *ERBB2*–RA Complex

**Figure 10:**
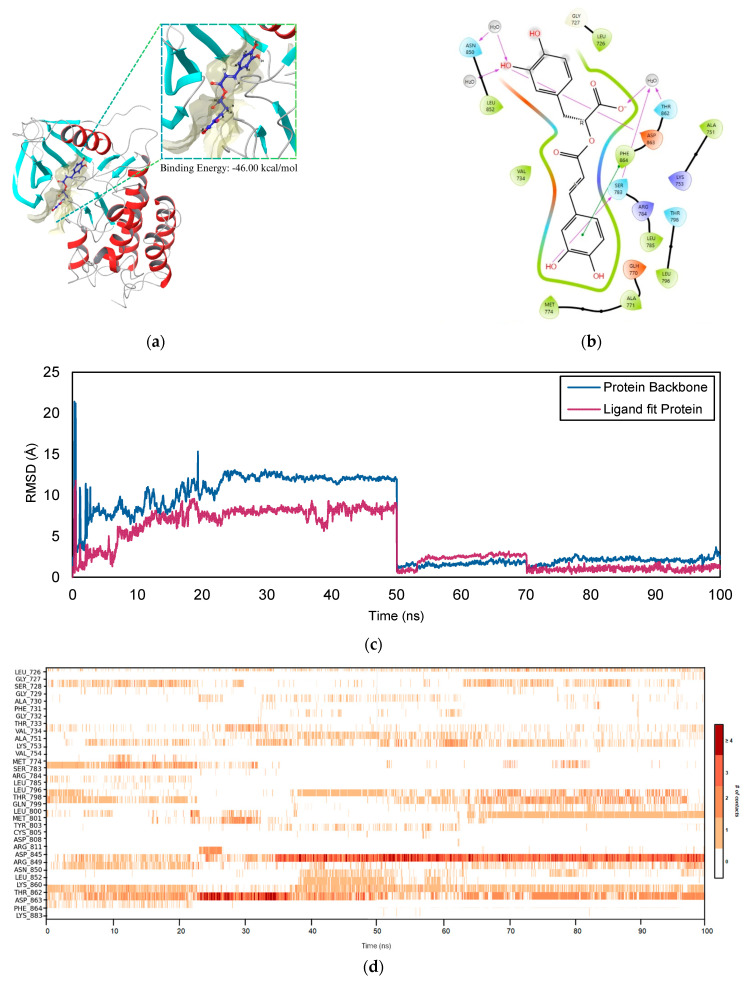
*ERBB2*–RA complex. (**a**) Docking pose of the ligand within the protein binding site with the calculated MM-GBSA binding energy reported in kcal/mol. (**b**) Two-dimensional interaction diagram illustrating detailed protein–ligand contacts. The stereochemical configuration with chiral centers assigned as (S) and (R) and double bonds designated as (E) and (Z). (**c**) RMSD plot of the protein–ligand complex over the 100 ns MD simulation, indicating structural stability. (**d**) Chronological map of protein–ligand contacts over the 100 ns MD simulation, depicting the persistence and dynamics of specific residue interactions throughout the trajectory. Residue contact frequency is represented by color, with darker shades of orange on the scale to the right indicating more frequent interactions.

The surface representing the binding site of the *ERBB2*–RA complex is depicted in Figure 10a. It has a binding energy of −46.00 kcal/mol, which suggests a high affinity between the interaction of its receptor and ligand. Figure 10b displays the 2D diagram representing the interaction between *ERBB2* and RA. The interaction between RA and the *ERBB2* receptor is highlighted in the 2D receptor–ligand complex diagram, which also displays important stabilizing factors. Hydroxyl groups and RA residues like THR 798 and SER 783 establish hydrogen bonds, and water molecules mediate further connections with LYS 753 and ASN 850 to increase stability. Hydrophobic interactions with residues such as LEU 785, PHE 864, and MET 774 further anchor the ligand within the binding pocket. In addition to π–π stacking with PHE 864, the complex is stable due to electrostatic interactions with negatively charged residues like ASP 863 and GLU 770. The interactions between the main chain and ALA 751, as well as LEU 726, contribute significantly to the overall binding strength. Water-mediated interactions and RA’s fit inside the *ERBB2* binding region highlight the high affinity of the receptor. These interactions indicate the importance of *ERBB2* in therapeutic applications for cancer treatment by pointing to possible inhibitory effects on its activity.

As seen in Figure 10c, the PL RMSD report for the *ERBB2*–RA complex over 100 ns is as follows. The *ERBB2*–RA complex initially undergoes incremental modifications, followed by more significant changes during the early phases of the simulation. At 10 ns, the protein backbone RMSD measures 2.399 Å, indicating minor structural changes as the protein adapts to RA. At this stage, the ligand RMSD is 2.273 Å, suggesting its initial mobility within the binding pocket. This phase demonstrates moderate flexibility in both the protein and ligand. A notable increase in RMSD values occurs between 30 ns and 50 ns. The protein RMSD rises from 2.527 Å at 30 ns to 2.653 Å at 50 ns, while the ligand RMSD experiences a significant jump from 3.485 Å to 8.485 Å. This increase indicates considerable reorientation or temporary destabilization of the ligand. These fluctuations suggest that the complex is exploring various conformations before settling into a stable state.

After 60 ns, the protein RMSD decreases to 1.166 Å, indicating that the system has stabilized, with minor fluctuations ranging from 1.057 Å to 2.427 Å. Concurrently, the ligand RMSD also drops to 0.979 Å at 60 ns and remains relatively stable, oscillating between 0.685 Å and 4.022 Å until the end of the simulation. This stability suggests that the *ERBB2*–RA complex has attained a well-defined binding conformation, characterized by minimal structural changes in the protein and a stabilized ligand position within the active site.

The interaction timeline for *ERBB2*–RA in Figure 10d shows inconsistent residue contacts. It can be observed that over 50 ns, ARG 849 and ASP 868 have been the main residues in contact with the ligand. As MD simulation progresses to 100 ns, the timeline exhibits stable and continuous contacts with ARG 849, ASP 863, MET 801, and TH862. Among these residues, frequent contacts were observed between the RA ARG 849 and ASP 863 via a combination of hydrogen bonding, hydrophobic contacts, and water bridges. Both residues were crucial as these play a major role in the stabilization of the complex as the MD simulation ends. The effect of these contacts can be seen from the low fluctuations of the reported RMSD.

Figure S11 presents the ligand RMSF analysis for the *ERBB2*–RA complex over 100 ns. The *ERBB2*–RA complex exhibits a broader range of RMSF values, from 1.45 to 2.59 Å, indicating that the RA ligand demonstrates flexibility. Notable atoms with elevated RMSF values are atom 5 (2.5856 Å), atom 25 (2.0417 Å), and atom 14 (2.0607 Å), indicating significant flexibility throughout the simulation. The elevated RMSF values indicate that these regions of the ligand exhibit high dynamics and may be exploring multiple conformations to optimize binding to the site or adapt to protein–protein interactions. The pronounced flexibility of RA implies that this ligand undergoes substantial structural rearrangements during the simulation, possibly indicating a more dynamic interaction with *ERBB2*. Specifically, the observed flexibility at atom 14 (2.0607 Å) and atom 25 (2.0417 Å) may reflect shifts in the functional groups of RA, potentially enhancing its binding affinity to the protein’s active site.

Furthermore, Figure S12 shows the analysis of the ligand properties of the *ERBB2*–RA complex. The rGyr of the *ERBB2*–RA complex exhibited significant variability throughout the 100 ns simulation, with values ranging from 46.16 Å to 51.79 Å. Initial equilibration and ligand binding modifications presumably cause the significantly enlarged conformation shown by the initial value of 51.79 Å at 10 ns. After that, this value dropped and settled between 46.16 Å and 47.49 Å by 40–60 ns, suggesting a more stable and compact pattern. Minor increases, like the peak at 48.31 Å at 80 ns, indicate a brief flexibility in the protein’s structure that solvent interactions or ligand dynamics may bring on. The overall decreasing trend indicates the stabilization of the complex as it transitions to a more compact form despite fluctuations.

Likewise, the MolSA demonstrated a less than 1.5% variance, fluctuating between 3219.21 Å^2^ and 3265.98 Å^2^. The lowest value, 3219.21 Å^2^ at 80 ns, indicates a brief constriction of the molecular surface. In contrast, the highest value of 3265.98 Å^2^ at 30 ns suggests a slight outward movement of surface residues during the initial equilibration phase. These minor variations do not indicate any significant disruptions to the size or structural integrity of the complex throughout the simulation.

In contrast, the SASA showed notable variability at 391.88 Å^2^ to 1848.43 Å^2^ (~370% fluctuation). The lowest value of 391.88 Å^2^, recorded at 10 ns, indicates a highly compact structure with minimal solvent exposure, likely due to strong interactions between the ligand and protein. Conversely, the peak value of 1848.43 Å^2^ at 50 ns suggests that SASA increased as the simulation progressed, reflecting a temporary conformational change that exposed hydrophilic residues to the solvent. Between 60 and 100 ns, SASA stabilized within a range of 1053.3 Å^2^ to 1593.51 Å^2^ following this peak, demonstrating the complex’s dynamic yet regulated solvent accessibility.

Throughout the simulation, the PSA varied, ranging from 3067.01 Å^2^ to 3209.18 Å^2^ (~5% variance). The highest value (3209.18 Å^2^ at 30 ns) indicates a brief increase in exposure to the polar area, which could improve interactions with the solvent through hydrogen bonds. In contrast, the lowest value (3067.01 Å^2^ at 20 ns) suggests a brief decrease in the exposure of polar residues, most likely due to conformational changes that buried certain polar areas. PSA maintained stability in the later phases despite these slight oscillations, confirming the preservation of crucial polar contacts necessary for complex stability.

#### 2.5.3. *TP53*–COH Complex

**Figure 11:**
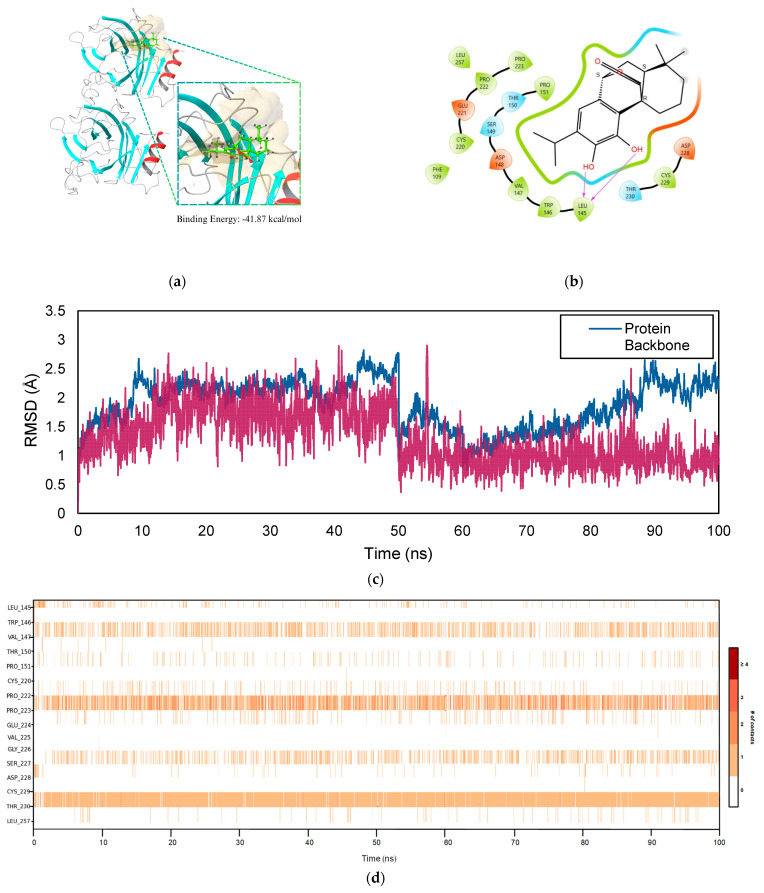
*TP53*–COH complex. (**a**) Docking pose of the ligand within the protein binding site with the calculated MM–GBSA binding energy reported in kcal/mol. (**b**) Two-dimensional interaction diagram illustrating detailed protein–ligand contacts. The stereochemical configuration with chiral centers assigned as (S) and (R) and double bonds designated as (E) and (Z). (**c**) RMSD plot of the protein–ligand complex over a 100 ns MD simulation, indicating structural stability. (**d**) Chronological protein–ligand contact map over a 100 ns MD simulation, depicting the persistence and dynamics of specific residue interactions throughout the trajectory. Residue contact frequency is represented by color, with darker shades of orange on the scale to the right indicating more frequent interactions.

Figure 11a demonstrates the surface characteristics of the complex binding site. The binding energy that was estimated for the *TP53*–COH complex is −41.87 kcal/mol. Figure 11b illustrates the 2D interaction diagram of the receptor–ligand complex between COH and the *TP53* protein. The ligand is anchored inside the binding pocket by hydrogen bonds that form between the hydroxyl groups on COH and LEU 145. Hydrophobic interactions with residues such as LEU 145, VAL 147, and TRP 146 further enhance stability. Negatively charged residues such as GLU 221 and ASP 148 interact with the polar areas of the ligand through electrostatic interactions. Main chain interactions with residues like SER 149 and THR 150 further enhance the stabilizing effects. Together with these interactions, the tight fit of COH in the *TP53* binding site indicates a high affinity for the protein. These findings highlight the possible modulatory effects of COH on *TP53*, which may possess therapeutic significance, especially in cancer treatment where *TP53* is crucial in controlling the cell cycle and apoptosis.

Figure 11c presents the PL RMSD analysis for the *TP53*–COH complex over 100 ns. The RMSD values for the *TP53*–COH complex indicate a distinct pattern of initial fluctuations followed by stabilization, akin to the behavior observed in the other complexes. At 10 ns, the protein backbone RMSD is 2.461 Å, indicating that the protein is undergoing an initial phase of conformational rearrangement while interacting with the ligand. The ligand’s RMSD begins at 1.642 Å, suggesting that the ligand is repositioning itself within the binding pocket, though the fluctuations observed at this point are relatively minor. From 20 ns to 50 ns, the protein and ligand slightly vary their RMSD values. The protein’s RMSD ranges from 1.507 Å at 20 ns to 2.698 Å at 50 ns, while the ligand fluctuates between 1.057 Å at 20 ns and 1.702 Å at 40 ns. Rather than indicating any significant structural alterations, these variations show that the protein and ligand are still acclimating to one another. The slight fluctuations suggest that the complex is progressing toward a stable configuration, though it has not completely attained it.

Figure 11d displays the chronological order map of protein–ligand contacts for the *TP53*–COH complex over 100 ns. Based on the figure, consistent and steady ligand–residue contacts occurred during the MD simulation. These residues were mainly SER 227, THR 230, PRO 223, and VAL 147. Among these residues, THR 230 has been reported to have the most frequent contact with the ligand through hydrogen bonding, which helps to anchor the ligand in the binding site. Aside from this, residue PRO 223 showed persistent contact via a combination of hydrophobic contacts, water bridges, and ionic interactions. These interactions contribute to the stability of the complex due to the steady contacts over 100 ns as the ligand remains tightly bound, which is reflected in a low and consistent RMSD profile.

Furthermore, the *TP53*–COH complex exhibits lower RMSF values, as illustrated in Figure S13, ranging from 0.58 to 1.06 Å, suggesting that the ligand maintains a relatively rigid and stable conformation throughout the simulation. Most of the atoms display low RMSF values, with atom 1 (0.8033 Å), atom 6 (0.7430 Å), and atom 18 (0.6177 Å) demonstrating the slightest fluctuations. The regions indicate that the ligand retains a stable structure while binding to *TP53*, reflecting robust and enduring interactions. Nonetheless, atoms 10 (1.0063 Å) and 16 (1.0353 Å) exhibit marginally more significant fluctuations, yet they stay within a relatively limited range, suggesting minor adjustments or localized flexibility in these areas. The limited flexibility observed suggests robust and positive interactions between COH and *TP53*, which probably play a significant role in the overall rigidity of the ligand. The consistent RMSF values for most of the ligand atoms in this complex indicate an optimized binding mode, implying that *TP53* and COH create a strong and stable complex with limited conformational alterations.

Additionally, Figure S14 shows the ligand characteristics of the *TP53*–COH complex. Between 3.995 Å and 4.051 Å, the rGyr of the *TP53*–COH complex showed very slight variations during the 100 ns simulation. The rGyr value of 4.051 Å at 10 ns first indicated a slightly enlarged conformation, most likely due to the early equilibration and modifications in the protein–ligand interaction. The rGyr value gradually declined throughout the simulation, ultimately stabilizing at approximately 3.995 Å between 90 and 100 ns. This trend suggests that the complex transitioned to a more compact and stable conformation. Transient increases, exemplified by the value of 4.022 Å at 60 ns, were noted; however, these fluctuations were minimal and fell within the anticipated range for flexible molecular systems. This observation underscores the relative stability of the protein, indicating that no significant structural changes occurred.

Concurrently, there was a minor variation of less than 2% in the MolSA values, ranging from 294.905 Å^2^ to 300.729 Å^2^. The slight conformational changes of the surface residues during the equilibration phase are probably responsible for the maximum MolSA value of 300.729 Å^2^, which was measured at 50 ns. At 80 ns, the lowest value, 294.905 Å^2^, shows a slight surface area contraction that may be related to the protein–ligand contact being even more stable. These slight changes show no notable structural disruptions during the simulation and that the complex retained the integrity of its molecular surface.

Conversely, the SASA demonstrated increased variability from 94.183 Å^2^ to 139.141 Å^2^, indicative of dynamic alterations in the protein–ligand complex. The minimal SASA value of 94.183 Å^2^ at 30 ns signifies a compact conformation with restricted solvent exposure, suggesting robust ligand binding. As the simulation continued, SASA rose and peaked at 139.141 Å^2^ at 60 ns, indicating that the complex experienced brief conformational changes that brought more of the structure into contact with the solvent. SASA then stabilized in the final phases of the simulation between 1053.3 Å^2^ and 1593.51 Å^2^, indicating the dynamic nature of the complex while maintaining overall stability.

The PSA fluctuated between 117.611 Å^2^ and 129.276 Å^2^ during the period of booting in, which was indicative of fluctuations in the exposure of polar regions. At 50 ns, the maximal PSA value of 129.276 Å^2^ suggests that polar residues are exposed to a greater extent, which may facilitate interactions with the solvent or ligand. The lowest value of 117.611 Å^2^ at 70 ns indicates a decrease in the exposure of polar regions, which is likely the result of conformational changes that concealed some of these residues. Despite these fluctuations, the PSA maintained a comparatively stable state throughout the latter portion of the simulation, highlighting the preservation of critical polar interactions crucial for the complex’s stability.

### 2.6. ADMET Analysis

Table 3 presents the molecular and physicochemical properties of COH, CA, and RA, which were analyzed to evaluate their drug-like potential. All three chemicals possess molecular weights that fall within the appropriate range for drug-like molecules—COH (330.423 g/mol), CA (332.439 g/mol), and RA (360.32 g/mol)—thereby providing favorable bioavailability. Hydrogen bonding and surface area study suggest that COH and CA, possessing 2–3 hydrogen bond donors and 3.5–4.5 acceptors, are more conducive to membrane permeability than RA, which has five donors and seven acceptors. Despite RA’s elevated solvent-accessible surface area (SASA) of 656.683 Å^2^ indicating enhanced solubility, it may lead to diminished permeability relative to CA (573.224 Å^2^) and COH (551.66 Å^2^).

Lipophilicity (LogP) and human oral bioavailability rates further distinguish the substances. COH (LogP = 2.991) and CA (LogP = 3.758) demonstrate ideal lipophilicity for membrane permeability, correlating with their elevated oral absorption rates of 100% and 89.53%, respectively. Conversely, RA (LogP = 1.213) exhibits more excellent hydrophilicity, resulting in a markedly reduced absorption rate of 38.34%, which may affect its bioavailability. Although all compounds adhere to Lipinski’s Rule of Five, suggesting potential for oral bioavailability, RA’s contravention of the Rule of Three underscores the constraints of fragment-based drug discovery. Given these results, it can be deduced that COH and CA exhibit a favorable balance of hydrogen bonding, lipophilicity, and absorption conducive to drug development. Despite its increased solubility, RA encounters difficulties owing to diminished permeability and bioavailability.

The toxicity predictions for COH, CA, and RA provide valuable insights into their safety profiles and potential biological effects, particularly concerning prostate cancer (Table 4). CA is identified as the most toxic among these compounds, with a predicted LD50 of 287 mg/kg, placing it in Toxicity Class 3 (highly toxic). It is expected to have activity related to cardiotoxicity and interact with cytochrome CYP3A4, indicating potential drug interactions that could complicate its use in combination therapies. Despite its toxicity concerns, CA does not engage significant pathways associated with prostate cancer, such as the androgen receptor (AR) and estrogen receptor (ER), suggesting that it may not directly influence prostate cancer progression through these receptors.

In contrast, COH shows moderate toxicity, with a predicted LD50 of 1500 mg/kg, classifying it as Toxicity Class 4. It demonstrates inactivity across several toxicity endpoints and metabolic pathways relevant to prostate cancer, including the Tox21 nuclear receptor signaling pathways encompassing androgen receptor binding. RA exhibits the most favorable safety profile, boasting a predicted LD50 of 5000 mg/kg (Class 5), indicating low toxicity. Like COH, RA shows inactivity concerning significant toxicity endpoints such as carcinogenicity, mutagenicity, and hepatotoxicity. Although RA is also inactive regarding androgen receptor binding, which is critical for prostate cancer therapy, its favorable toxicity profile makes it a safer compound overall.

All three compounds are expected to cross the blood–brain barrier, which may affect central nervous system effects. However, since none of the compounds exhibits significant activity on androgen receptor signaling, their direct therapeutic potential for prostate cancer remains limited. Overall, CA is identified as the most toxic compound, while RA shows the least impact on prostate cancer pathways, positioning it as the most promising candidate for further research.

## 3. Discussion

Studies have been conducted regarding the possible anti-PCa effects and bioactive compounds of rosemary, demonstrating growth-inhibiting properties in PCa cells [46]. It has been reported that rosemary extracts have high concentrations of polyphenols such as RA, CA, and COH, which are suggested to have antioxidant and anticancer properties [47,48,49,50,51]. These substances may be employed as therapeutic substitutes in managing and preventing PCa [20]. Furthermore, natural compounds made from edible plants have a more comprehensive range of targets than synthetic compounds, which makes them desirable options for anti-PCa medications [52]. Previous studies show the effect of RA in reducing cell growth and inducing apoptosis in PC-3 and DU-145 PCa cell lines [53]. Significantly, COH has been reported to increase apoptosis, cause cell cycle arrest, and suppress cell growth in PC3 cells [54]. Important signaling pathways including PI3K/Akt and AMPK are also modulated by it [55]. Moreover, CA has been found to provide a potential treatment approach for PCa by facilitating AR degradation through ER stress [56]. It modifies critical signaling pathways to prevent PCa cell lines, such as DU-145 and PC3, from proliferating and to cause them to undergo apoptosis [57].

Rosemary extracts and active metabolites were tested on various human cancer cell lines using in vitro assay evaluation. Existing studies indicate that the plant extract showed cytotoxic effects against different PCa cell lines PC3 and DU-145, inhibiting cell viability [47]. Regardless of being a cell line that is negative for AR expression, recent findings indicate that DU-145 exhibits low to moderate levels of positive AR expression compared to other cell lines expressing the AR [58]. The essential inhibitory effects on sensitive and insensitive androgen cell lines reveal possible chemotherapeutic effects on various subtypes of PCa [59]. Given this, the results obtained from the present study show the dose–response behavior of water and ethanol rosemary extracts more evidently at higher concentrations of the extract. The evident response of DU-145 cells to RL-W at high doses revealed that a lower concentration was required to result in 50% cytotoxicity in DU-145 cells. Findings from this in vitro study imply that rosemary leaf extract and its active compounds have potential anticancer properties against PCa. Nonetheless, it is crucial to validate the therapeutic effects of metabolites on PCa progression through molecular docking based on the conducted network pharmacology analysis using relevant target genes involved in PCa cytotoxicity.

The biological mechanisms that regulate the action of several herbal compounds in the medical treatment of PCa have been explored using network pharmacology and molecular docking techniques. Several investigations delved into the possible targets and pathways associated with the cancer-fighting properties of various herbal remedies [60,61,62,63,64]. In this study, the enrichment analysis of 37 overlapping target genes revealed significant involvement in biological processes and pathways critical to PCa progression. GO terms related to biological regulation, protein binding, cell proliferation, and metabolic processes were prominently enriched, reflecting the complex regulatory networks that drive prostate tumor growth and metastasis. Significantly, pathways such as insulin-like growth factor receptor signaling and tyrosine metabolism have been implicated in PCa development and prognosis, highlighting the role of metabolic reprogramming and signal transduction dysregulation in disease progression [43,65,66]. Metabolic alterations, including changes in phosphate metabolic processes and non-membrane-spanning protein tyrosine kinase activity, further underscore the dynamic cellular environment of prostate tumors [67,68,69].

The KEGG pathway enrichment analysis revealed that MAPK, PI3K/AKT, calcium signaling, and central carbon metabolism pathways—commonly active in AR-negative PCa—contribute to severity [70,71]. Aside from this, it was revealed in the report that several genes are known to promote tumor growth and survival independently of AR signaling. Moreover, the Reactome pathway analysis identified enrichment in PI3K/AKT signaling and activation of matrix metalloproteinases, pathways known to facilitate cancer cell survival, proliferation, invasion, and metastasis [69]. Dysregulation of the PI3K/AKT pathway, often driven by mutations in components such as PIK3R1, is associated with increased glycolysis and poor clinical outcomes in metastatic prostate cancer [67]. Matrix metalloproteinases, including MMP9, contribute to extracellular matrix remodeling, promoting tumor invasion and dissemination [72]. Given these findings, the enrichment of AR-independent pathways supports investigating bioactive compounds of rosemary as potential multi-targeted therapies to inhibit tumor growth and survival in AR-negative prostate cancer, where conventional hormonal treatments are ineffective.

The identified targets in this study included essential genes in PCa: *EGFR*, *TP53*, *ERRB2*, *IGFBP3*, *MMP-2*, *HDAC6*, *FGFR1*, *MMP-9*, and *PDGFRB*. A crucial hub gene for PCa is *TP53*, which is overexpressed in PRAD, and the status of *TP53* mutations has been demonstrated to stratify PCa, leading to variations in the molecular mechanism [73,74]. By stimulating the AR, *EGFR* and *ERBB2* contribute to the growth of PCa in hormone-deficient environments [75]. PCa cells have been established to become more susceptible to apoptosis during androgen withdrawal therapy (AWT) due to the dual inhibition of EGFR and HER2 [76]. Advanced PCa is linked to abnormal spatial patterns of *EGFR*, including increased diffusivity, activation, migration, proliferation, and invasion [77]. *HDAC6* is involved in increasing cancer cell proliferation and the stress response, whereas *MMP2* has been linked to cancer cell invasion and a poor prognosis. In comparison with single-target inhibitors, dual-target inhibitors, which target both *MMP2* and *HDAC6*, demonstrated the potential to increase therapeutic response rates [78,79,80].

In addition, research has shown that tumors with *FGFR1*, a receptor tyrosine kinase that has been implicated in various cancers, have worse differentiation and are more likely to spread, whereas tumors lacking it have smaller and more differentiated phenotypes [81]. PCa frequently exhibits elevated *MMP-9* expression, which promotes metastasis by allowing tumor cells to penetrate through the bloodstream through the basement membrane [82]. The receptor tyrosine kinase *PDGFRB* is also implicated in angiogenesis and cell proliferation. *PDGFRB* signaling drives smooth muscle cells and pericytes to newly created blood vessels, which aids in angiogenesis and encourages tumor growth by promoting cell survival and proliferation [83,84]. The progression of PCa is likely influenced by the interplay of these targets where *FGFR1* drives tumor growth and metastasis, *MMP-9* facilitates invasion, and *PDGFRB* promotes tumor growth and angiogenesis. Apart from that, *IGFBP3* contributes to the increasing incidence of PCa, is associated with unfavorable health outcomes, and is overexpressed in cell lines immune to Olaparib [85,86,87]. In resistant models, inhibition of *IGFBP3* increases DNA double-strand breaks and cell death, and it is suggested that the *IGFBP3/EGFR* signaling axis involved in modulating DNA repair may be connected with PARP inhibitor resistance [88,89].

The in silico study on rosemary was conducted by evaluating the MM-GBSA binding energy and molecular dynamics from the interactions with RA, CA, COH, 5-FU, and designated reference drug with the affected genes from the PPI network and KEGG analysis. The MM-GBSA binding energy provides an estimate of the binding affinity between a ligand and a protein, wherein a more negative value indicates strong binding [90,91]. The re-docking approach has been validated, indicating its reliability and precision in predicting protein targets. Strong binding of COH and RA to *TP53*, *IGFBP3*, and *HDAC6* suggests that they may be promising treatment prospects. Additionally, their binding energy for EGFR is competitive, indicating a high level of affinity for this target. Most targets show less favorable binding for CA, whereas 5-FU has moderate binding energies. Strong affinity is indicated by the proteins in complexes with reference drugs having the largest negative binding energies. These findings suggest that COH and RA are promising therapeutic candidates for further investigation, particularly for targets like *HDAC6* and *TP53*.

The molecular dynamics (MD) simulations of three receptor–ligand complexes—*EGFR*–COH, *ERBB2*–RA, and *TP53*–COH—were conducted based on their relevance to biological pathways, prominence in protein networks, and estimated binding energies. These simulations revealed varying levels of stability and ligand flexibility among the complexes. The stability of protein–ligand interactions is crucial and is indicated by low RMSD values, which signify minimal deviations from the original structure [92,93]. The *TP53*–COH complex exhibited the highest structural rigidity and stability, with extremely low RMSF values, suggesting a rigid and stable ligand conformation due to strong protein–ligand interactions [57]. In contrast, the *ERBB2*–RA complex showed higher ligand fluctuations, indicating more dynamic interactions. The *EGFR*–COH complex demonstrated stable binding with minimal ligand variations, maintaining the binding contact with minor fluctuations.

These complexes achieved stable conformations by the end of the simulation, with the *TP53*–COH complex showing the highest structural rigidity. The receptor–ligand interactions were robust and maintained by electrostatic contacts, hydrophobic interactions, and hydrogen bonds. The stability of protein–ligand complexes, as indicated by low RMSD values, is crucial for maintaining strong interactions between proteins and ligands. Strong interactions including hydrogen bonds and hydrophobic contacts, which are seen in the *EGFR*–COH and *TP53*–COH complexes, frequently support this stability [94,95]. On the contrary, the *ERBB2*–RA complex fluctuates, indicating the flexibility of the ligand inside the site of binding, which may influence binding stability and affinity. Interactions such as hydrogen bonding and π–π stacking keep the strong affinity of these complexes intact. In this regard, RA engages in π–π stacking with *ERBB2*, whereas COH creates hydrogen bonds with *EGFR* and *TP53* [96,97]. By modifying important pathways implicated in the genesis of cancer, these interactions highlight the therapeutic potential of COH and RA as anti-PCa agents.

Importantly, this study highlights the potential of *Rosmarinus officinalis* L. extracts in targeting androgen receptor (AR)-independent pathways in prostate cancer. Although DU-145 cells are often classified as AR negative or androgen insensitive, conventional treatments such as androgen deprivation therapy (ADT) remain ineffective in this subtype. Our network pharmacology analysis identified key targets—including *EGFR, ERBB2*, *TP53*, *HDAC6*, *FGFR1*, *MMP9*, and *PDGFRB*—that are well known for driving prostate cancer progression independent of AR signaling. These findings were further validated by molecular docking and MM-GBSA binding energy calculations, where compounds such as carnosol and rosmarinic acid showed strong affinities for AR-independent targets (e.g., *TP53*: −41.87 kcal/mol; *ERBB2*: −46.00 kcal/mol). KEGG and Reactome enrichment analysis confirmed that the identified targets are significantly involved in pathways such as PI3K/AKT, MAPK, calcium signaling, and cell communication—mechanisms commonly active in AR-negative PCa. This multi-target approach contrasts with AR-focused therapies and may offer therapeutic advantages, particularly in treatment-resistant prostate cancer subtypes.

The molecular characteristics, bioavailability, and safety profiles of COH, CA, and RA for the treatment of prostate cancer were assessed using ADMET analysis. CA and COH exhibit favorable drug-like characteristics with moderate lipophilicity and high oral absorption, adhering to RO5 criteria. However, CA shows high toxicity, particularly cardiotoxicity, limiting its therapeutic potential. COH displays moderate toxicity but remains inactive in most toxicity endpoints. In contrast, RA, despite lower bioavailability, presents a favorable safety profile with minimal toxicity, making it a promising candidate for further research in prostate cancer treatment due to its low risk of side effects.

The potential anti-PCa properties of RL and its metabolite offer a promising approach to treating and preventing the advancement of PCa. Accordingly, additional in vitro and in vivo research is required to test and contrast the effects of RL on various PCa cell lines. The role of RL active compounds in therapeutic approaches on pertinent pathways involving PCa and tumor progressions has been highlighted using molecular docking, MM-GBSA calculation, MD simulation, and ADMET analysis. Research conducted in vitro and in silico has yielded favorable results, suggesting that RL may be a viable candidate for future drug development aimed at combating PCa.

## 4. Materials and Methods

### 4.1. Water and Ethanol Extractions of Rosemary Leaves

This study used two solvent extracts: water (RL-W) and ethanol (RL-E) extracts. Leaves of rosemary were dried. In a decoction pot, RL-W was prepared by adding 50 g of dried rosemary leaves and 1000 mL of double-distilled (D.D.) water until the solution was reduced to about 200 mL. On the other hand, 50 g of dried rosemary leaves and 100 mL of 95% ethanol solvent were placed in a round bottom flask and shaken to prepare RL-E. This flask was then subjected to reflux and a water bath at 65 °C for two hours with frequent shaking. These extract solutions were both filtered using vacuum filtration. Subsequently, filtered extract solutions were subjected to rotary evaporation, in which the water bath heaters for RL-W and RL-E were set to 60 °C to 70 °C and 50 °C, respectively. The speed of the rotary evaporator was set to a value of 3 rps. After this, these were placed in a freeze-dryer until all sample extracts were dried.

### 4.2. Liquid Chromatography-Mass Spectrometry (LC-MS) Analysis

High-resolution and high-mass accuracy LC-MS experiments were performed on an LTQ-FT ultra mass spectrometer (Thermo Electron, San Jose, CA), which combines a linear quadrupole ion trap with Fourier transform ion cyclotron resonance (FT-ICR). The instrument was equipped with a standard electrospray ionization (ESI) source, an Agilent 1100 Series binary high-performance liquid chromatography (HPLC) pump (Agilent Technologies, Palo Alto, CA, USA), and a Famos autosampler (LC Packings, San Francisco, CA, USA). Samples (5 μL) were injected into an Acquity UPLCpeptide CSH C18 column (1 mm I.D. × 150 mm, 1.7 µm, 130 Å). Chromatographic separation was achieved using 0.1% formic acid in water as mobile phase A and 0.1% formic acid in acetonitrile as mobile phase B, with a flow rate of 50 μL/min. The gradient elution program was as follows: 2% mobile phase B at 2 min, increasing to 98% mobile phase B at 60 min. Electrospray ionization was performed at a voltage of 3.0 kV, and the capillary temperature was set to 275 °C. Mass spectrometric analysis was conducted in the m/z range of 100–1000 using the FT-ICR mass analyzer at a resolution of 100,000 (at m/z 400).

### 4.3. Anti-Prostate Cancer Activity of Rosemary Leaf Extracts

#### 4.3.1. Cell Culture

Human PCa DU-145 cells, obtained from Bioresource Collection and Research Center (BCRC, Taiwan), were cultured in Eagle’s Minimum Essential Medium (EMEM) prepared with 10% fetal bovine serum (FBS) and 1% penicillin-streptomycin in a standard-sized petri dish [98]. DU-145 cells were incubated at 37 °C with 5% CO_2_ for two days. Sub-culture was performed on these cells within two to three days once cell confluence observed under the microscope had reached approximately 80%.

#### 4.3.2. Cell Treatment

DU-145 cells at a concentration of 6 × 10^5^ cells/well were seeded in a 96-well microarray plate. The seeded plate was incubated for two nights at 37 °C. Cell treatments were carried out using controls and varying plant extract concentrations. A 100 mg/mL stock solution of each crude extract was prepared by dissolving RL-W with deionized water and RL-E with 5% ethanol. Briefly, 10 mg/mL sample treatment media containing 100 μL stock solution diluted with 900 μL medium was prepared and filtered using a 0.2 μm syringe filter. Varying concentrations of plant extract treatments were obtained with two-fold serial dilution from 10 to 0.625 mg/mL. The positive control in the experiment was 1 mg/mL 5-fluorouracil (5-FU), whereas the negative control was only the medium. These controls and treatment media were added to each well for a 24-h treatment period in an incubator at 37 °C with 5% CO_2_. Triplicate trials for each treatment and control group were observed to validate the results.

#### 4.3.3. MTT Cell Viability Assay

An MTT reagent with a concentration of 5 mg/mL was prepared by dissolving thiazolyl blue tetrazolium bromide (approx. 98% TLC) in phosphate-buffered saline (PBS) under pH 7.4 and was stored at 4 °C protected from light. The reagent was warmed at 37 °C before the MTT cell viability assay was performed. Treatment media from the 96-well microarray plate were aspirated, and each well was carefully washed using 100 μL to ensure serum and phenol red were removed. Following this, 90 μL PBS and 10 μL MTT reagent were added to each well, and the plate was incubated for 4 h at 37 °C with 5% CO_2_. Formazan crystals formed on each well were dissolved in a 100 μL solubilizing buffer dimethyl sulfoxide (DMSO) for 15 min after removing the PBS and MTT reagent. Absorbances at 570 nm and a reference absorbance of 650 were measured using a Thermo Fisher Mulyiskan SkyHigh Microplate Spectrophotometer (Thermo Fisher Scientific Inc., Waltham, MA, USA) [99].

### 4.4. Data Treatment and Statistical Analysis

Microsoft Excel was utilized for the data processing, and data are reported as the mean with calculated standard deviation. Consequently, these experimental data were statistically analyzed using GraphPad Prism 9 (GraphPad Software, Inc., Boston, MA, USA) by applying two-way repeated measure ANOVA with replication with a family-wise alpha threshold and confidence value of 0.05 (95% confidence interval). Similarly, the same software was used to visualize the cytotoxicity percentage of the treatment and controls.

### 4.5. Network Pharmacology

#### 4.5.1. Screening and Identification of Gene Targets

Gene Expression Profiling Interactive Analysis 2 (GEPIA 2, http://gepia2.cancer-pku.cn/#, accessed on 21 October 2024) and Gene Expression Omnibus (GEO, https://www.ncbi.nlm.nih.gov/geo/, accessed on 21 October 2024) with GEO accession GSE179324, considering only DU-145 cell lines from the data, were used in this study [100,101,102]. Values set for the |Log2FC| cutoff and q-value cutoff were 1 and 0.01, respectively [98]. The differential method chosen was LIMMA to compare differentially expressed genes of PRAD versus normal prostate cells. Meanwhile, identified metabolite gene targets were obtained from SwissTargetPrediction (http://www.swisstargetprediction.ch/, accessed on 21 October 2024) [103]. The shared gene set was determined from BioTools Venn Diagrams (https://www.biotools.fr/misc/venny, accessed on 21 October 2024).

#### 4.5.2. Protein–Protein Interaction Network Construction

Intersected genes obtained from the BioTools Venn Diagram were input in STRING (https://string-db.org/, accessed on 21 October 2024), increasing the interaction score to 0.900 (highest confidence) [104]. The PPI network was sent to Cytoscape version 3.0. Centiscape for further analysis using cytoHubba and cytoNCA plugins [32,105,106]. Proteins were ranked according to Betweenness (BC), Closeness (CC), Maximal Clique Centrality (MCC), Density of Maximum Neighborhood Component (DMNC), and Degree (DC), and the top six proteins were selected for further molecular docking.

#### 4.5.3. Gene Function and Pathway Enrichment Analysis

WebGestalt (https://www.webgestalt.org/, accessed on 2 November 2024) was used for the enrichment analysis of Gene Ontology (GO) and pathway data [41,107,108]. The GO slim summary was used to visualize the GO enrichment analysis. Potential gene target symbols for Homo sapiens were input into WebGestalt, and the method of interest used was Over-Representation Analysis (ORA). The parametric significance level (FDR) was set to 0.05 for the analysis. Visual representations on KEGG pathways were obtained from ShinyGO (http://bioinformatics.sdstate.edu/go/, accessed on 2 November 2024), whereas Reactome pathways were obtained from the Reactome Pathway Database (https://reactome.org/, accessed on 2 November 2024) [107,109,110,111].

### 4.6. Molecular Docking

#### 4.6.1. Docking Validation

The identified protein targets were subjected to molecular docking analysis using Schrödinger Maestro 13.5 (Schrödinger, Inc., New York, NY, USA) to calculate binding energies and evaluate the interactions between the receptor–ligand complexes. Crystal structures of selected target proteins were collected from the RCSB Protein Data Bank (RCSB PDB) (https://www.rcsb.org/, accessed on 13 November 2024) [112]. Respective PDB IDs of proteins with co-crystallized ligands were selected from UniProt (https://www.uniprot.org/, accessed on 13 November 2024) and the existing literature on molecular docking [53,113,114,115,116,117]. Each PDB ID was imported directly into the Protein Preparation Workflow, where it was preprocessed, including the deletion of water molecules beyond 5.00 Å from the protein structure. Following this, diagnostics were performed to ensure that the significant protein chain and any co-crystallized ligand remained intact. Overlapping hydrogens were addressed through optimization using PROPKA, after which the structure was minimized, and additional water molecules were removed. The glide grid of the protein was obtained using the Receptor Grid Generation function from the Glide application. The co-crystal ligand was selected from the prepared protein. Under the Site tab, the centroid of the workspace ligand was chosen to define the enclosing box. In the Advanced Settings, the dimensions of the box in the x, y, and z coordinates were adjusted to ensure the ligand was properly enclosed within the site where the co-crystallized ligand was bound.

Next, the co-crystallized ligand was extracted from the prepared protein structure and then prepared using LigPrep at pH 7.00 ± 2, with Epik and the OPLS4 force field. The ligand was subsequently subjected to a Ligand Docking task, where the resulting Glide grid file was used for the receptor grid. The docking precision and ligand sampling were set to XP (extra precision) and flexible in the Settings tab to optimize the docking process. The Superposition tool was employed to validate the docking approach. In this step, the docked ligand and the reference co-crystal ligand structures were superimposed, and the corresponding RMSD value was calculated. The RMSD values were compared to the accepted threshold of less than 2.0 Å.

#### 4.6.2. Receptor–Ligand Docking

Three-dimensional (3D) conformer structured data files (SDF) of ligands were obtained from PubChem (https://pubchem.ncbi.nlm.nih.gov/, accessed on 14 November 2024) [118]. The 3D structures of these ligands were prepared using LigPrep at pH 7.00 ± 2 with Epik, generating at most one conformer per ligand. The selected force field was OPLS4. Afterward, the docking process was performed using these metabolites and reference drugs with the Glide grid from the validated docking approach for the binding site. Alternatively, a blind docking approach was applied for proteins with limited PDB structures, as they lack available co-crystallized ligands. The SiteMap tool was employed to identify, visualize, and evaluate protein binding sites to generate a Glide grid for the docking analysis. Each ligand was docked using Glide with the generated grids, applying XP precision and flexible ligand sampling to generate receptor–ligand complexes. The binding site with the most negative binding energy was used.

#### 4.6.3. Molecular Mechanics Generalized Born Surface Area (MM-GBSA) Calculations

Following the completion of the docking process, MM-GBSA analysis was conducted to refine the binding affinity predictions and provide a more accurate estimation of the binding free energy of the ligand–protein complexes. The Prime MM-GBSA method was specifically used to calculate the binding energy of each ligand–protein complex that had been generated during the molecular docking simulations. The estimated binding energy for each complex was carefully analyzed to assess the strength and stability of the binding interaction. By evaluating the binding energies, complexes with the most favorable binding affinities were identified, helping to pinpoint which ligand–protein pairs demonstrated the most stable and energetically favorable interactions.

### 4.7. Molecular Dynamics Simulation

A molecular dynamics (MD) simulation was conducted to evaluate the stability, flexibility, and interaction dynamics of the receptor–ligand complex under physiological conditions. To simulate the selected complexes, System Builder and Molecular Dynamics tools from Desmond (Schrödinger, Inc., New York, NY, USA) were utilized with the OPLS4 force field. Before the generation of the model system and the execution of the MD simulation, the complexes were carefully prepared. In the System Builder, the solvent model was set to Simple Point Charge (SPC), and the boundary conditions were configured to an orthorhombic box shape. The buffer box size was determined using a predefined calculation method. The dimensions of the box (distances for a, b, and c) were set to 10.0 Å to ensure proper spacing between the molecules, after which the system volume was minimized to reduce any initial steric clashes. To ensure that the system was electrically neutral, recalculated ions were added to counteract any charge imbalances. Additionally, salt was incorporated to mimic physiological ionic strength, which is essential for maintaining the correct electrostatic environment for accurate simulations. These steps were carefully performed to prepare the system for the subsequent MD simulation, ensuring that the receptor–ligand complex behaves realistically under simulated physiological conditions.

Following this preparation, the model system was used in the Molecular Dynamics tool, where the simulation time was set to 100 ns under constant conditions of 300.0 K and 1.01325 bar. The simulation was run for 2–3 days, depending on the size of the complex and the number of atoms involved. At the end of the simulation, the resulting output files were loaded into the Simulation Interaction Diagram (SID) tool for detailed analysis and visualization by providing a comprehensive report on various properties of the system. In this analysis, key metrics such as root mean square deviation (RMSD), root mean square fluctuation (RMSF), and ligand properties including radius of gyration (Rgyr), solvent-accessible surface area (SASA), polar surface area (PSA), and molecular surface area (MOLSA) were used to evaluate the structural stability, conformational dynamics, and interactions of the selected complexes.

### 4.8. Absorption, Distribution, Metabolism, Excretion, and Toxicity (ADMET) Analysis

ADMET analysis was conducted to predict the absorption, distribution, metabolism, excretion, and toxicity profiles of the studied compounds, providing insights into their pharmacokinetic properties and the drug likeness of rosemary metabolites. The structures of the ligands of interest were used in the QikProp tool of Maestro, in which relevant molecular descriptors were calculated to assess their ADMET properties. Furthermore, additional toxicity analysis was executed in ProTox 3.0 (https://tox.charite.de/protox3/index.php?site=home, accessed on 26 November 2024) to assess the potential toxicological profiles of the ligands [119]. ProTox 3.0 provided predictions on various toxicity models. These findings, combined with the results of the in vitro experiments, helped refine the ADMET analysis.

## 5. Conclusions

Rosemary is widely recognized for its medicinal properties, and its extract metabolites have been studied for their potential in prostate cancer treatment. However, the evaluation of these metabolites in AR-negative cell lines, such as DU-145, remains an area that warrants further investigation. The in vitro evaluation of the cytotoxic effects of rosemary against DU-145 cells in the present study shows the dose–response behavior of extracts to inhibit PCa cell survival with varying concentrations. The half-maximal cytotoxic concentration (IC_50_) of rosemary aqueous extract suggests that the water solvent system highly inhibits DU-145 cell survival. The in silico study provides a comprehensive framework for understanding the therapeutic potential of rosemary bioactive compounds, particularly rosmarinic acid and carnosol, in PCa treatment. By employing molecular docking, MM-GBSA calculations, MD simulations, and ADMET analysis, the research highlights the significant roles these metabolites play in drug discovery and development. Rosmarinic acid and carnosol demonstrate strong binding affinities and favorable pharmacokinetic properties, suggesting their potential as anti-PCa agents. Furthermore, the study emphasizes that rosemary leaf extracts derived from water and ethanol could serve as viable therapeutic agents against PCa. This paper lays a foundation for future research involving in vitro and in vivo studies to further explore the mechanisms of action of rosemary extracts. Such investigations could deepen the understanding of their role in PCa treatment while advancing their development as chemotherapeutic agents.

## Figures and Tables

**Table 1:** Validation of the docking approach for PCa target proteins.

Receptor	PDB ID	Co-Crystallized Ligand ID	RMSD (Å)
*EGFR*	1M17	AQ4	1.2451
*ERRB2*	3PP0	03Q	0.6354
*FGFR1*	7WCL	8ZF	1.7018
*HDAC6*	5EDU	TSN	0.7883
*MMP-2*	1HOV	I52	1.3172
*MMP-9*	1GKC	NFH	1.4014
*PDGFRB*	3MJG	NDG	1.3752
*TP53* (Y220C Mutation)	2VUK	P38	0.3593
*IGFBP3* ^1^	7WRQ	-	-

^1^ Blind docking approach.

**Table 2:** Receptor–ligand MM-GBSA binding energy (kcal/mol).

	*EGFR*	*ERBB2*	*FGFR1*	*HDAC6*	*MMP-2*	*MMP-9*	*PDGFRB*	*TP53*	*IGFBP3* ^3^
COH	−35.04	−14.13	−37.11	−49.41	−25.91	−30.05	−29.83	−41.87	−42.85
CA	−24.93	4.69	−8.52	−21.02	13.28	−25.77	−8.44	−29.8	−16.08
RA	−30.36	−46.00	−30.22	−38.12	−39.77	−42.28	−35.27	−34.47	−47.23
5-FU ^1^	−20.43	−21.09	−23.73	−28.67	−22.97	−31.15	−16.65	−16.48	−22.28
RD ^2^	−55.75	−86.05	−86.12	−44.46	−67.65	−78.27	−36.18	−49.77	-

^1^ In vitro standard drug; ^2^ In silico reference drugs [(Erlotinib, TAK-285, Pemigatinib, Trichostatin A, SC-74020, N~2~-[(2R)]-2-{[formyl(hydroxy)amino]methyl}-4-methylpentanoyl]-N,3-dimethyl-L-valinamide, Phikan083, and Crenolanib, respectively); ^3^ Blind docking approach.

**Table 3:** Molecular and physicochemical properties of carnosol, carnosic acid, and rosmarinic acid.

	Carnosol	Carnosic Acid	Rosmarinic Acid
Molecular Weight	330.423	332.439	360.32
Hydrogen Bond Donors	2	3	5
Hydrogen Bond Acceptors	4.5	3.5	7
Solvent Accessible Surface Area (SASA)	551.66	573.224	656.683
Octanol-Water Partition Coefficient (LogP)	2.991	3.758	1.213
Percent Human Oral Absorption (%)	100	89.53	38.34
Lipinski’s Rule of Five (RO5)	0	0	0
Rule of Three (RO3)	0	0	1

**Table 4:** Summarized toxicity model report from ProTox 3.0. The more intense color indicates a higher probability of binding to the toxicity target.

Toxicity Category	Specific Toxicity or Pathway	Prediction and Probability		
		Carnosol	Carnosic Acid	Rosmarinic Acid
Organ Toxicity	Hepatotoxicity	Inactive (0.76)	Inactive (0.63)	Inactive (0.62)
	Cardiotoxicity	Inactive (0.54)	Active (0.56)	Inactive (0.69)
Toxicity End Points	Carcinogenicity	Inactive (0.62)	Inactive (0.60)	Inactive (0.66)
	Mutagenicity	Inactive (0.88)	Inactive (0.84)	Inactive (0.85)
	BBB Barrier	Active (0.67)	Active (0.65)	Active (0.62)
Tox21-Nuclear Receptor Signaling Pathways	Androgen Receptor (AR)	Inactive (0.96)	Inactive (0.87)	Inactive (0.94)
	Estrogen Receptor Alpha (ER)	Inactive (0.74)	Inactive (0.64)	Inactive (0.74)
	PPAR-Gamma	Inactive (0.94)	Inactive (0.94)	Inactive (0.80)
Tox21-Stress Response Pathways	Nrf2/ARE	Inactive (0.81)	Inactive (0.80)	Inactive (0.88)
	Phosphoprotein (Tumor Suppressor) p53	Inactive (0.78)	Inactive (0.82)	Inactive (0.73)
Metabolism	Cytochrome CYP3A4	Inactive (0.65)	Active (0.73)	Inactive (0.94)
	Cytochrome CYP2E1	Inactive (1.0)	Inactive (1.0)	Inactive (1.0)
Oral Toxicity	Predicted LD50 (mg/kg)	1500	287	5000
	Predicted Toxicity Class	4	3	5

## Data Availability

All relevant data are within the paper.

## Supplementary Materials

The following supporting information can be downloaded at: https://www.mdpi.com/article/10.3390/ijms26104650/s1.
