# Supplementary material for: In Vitro and In Silico Evaluation of the Potential Anti-Prostate Cancer Activity of Rosmarinus officinalis L. Leaf Extracts
Source: Int J Mol Sci. 2025 May 13;26(10):4650. doi: 10.3390/ijms26104650 (PMC12111643; doi:10.3390/ijms26104650)
Supplement: Supplementary file 1 [file ijms-26-04650-s001.zip › ijms-3624638-supplementary.pdf]

SUPPORTING INFORMATION

ABSORBANCE READINGS FROM THE MTT ASSAY

Table S1. Absorbance readings at 570 nm

Sample Concentration, mg/mL		10	5	2.5	1.25	0.625	Positive Control[a]	Negative Control[b]
Water Extract	R1	0.058	0.059	0.106	0.399	0.467	0.149	0.612
	R2	0.050	0.052	0.144	0.394	0.536	0.091	0.651
	R3	0.053	0.053	0.163	0.367	0.522	0.140	0.719
Ethanol Extract	R1	0.056	0.051	0.187	0.402	0.471	0.149	0.549
	R2	0.060	0.050	0.206	0.529	0.457	0.091	0.629
	R3	0.059	0.051	0.168	0.405	0.466	0.140	0.537

[a] 1 mg/mL 5-Fluoruracil
[b] DU-145 cells without sample dosage

Table S2. Absorbance readings at 650 nm

Sample Concentration, mg/mL		10	5	2.5	1.25	0.625	Positive Control[a]	Negative Control[b]
Water Extract	R1	0.045	0.046	0.05	0.072	0.087	0.055	0.1020
	R2	0.041	0.045	0.054	0.073	0.089	0.058	0.0955
	R3	0.045	0.046	0.045	0.071	0.089	0.067	0.0975
Ethanol Extract	R1	0.044	0.043	0.057	0.077	0.086	0.055	0.0459
	R2	0.046	0.042	0.058	0.089	0.088	0.058	0.0474
	R3	0.044	0.043	0.055	0.079	0.088	0.067	0.0458

[a] 1 mg/mL 5-Fluoruracil
[b] DU-145 cells without sample dosage

PROTEIN-PROTEIN NETWORK

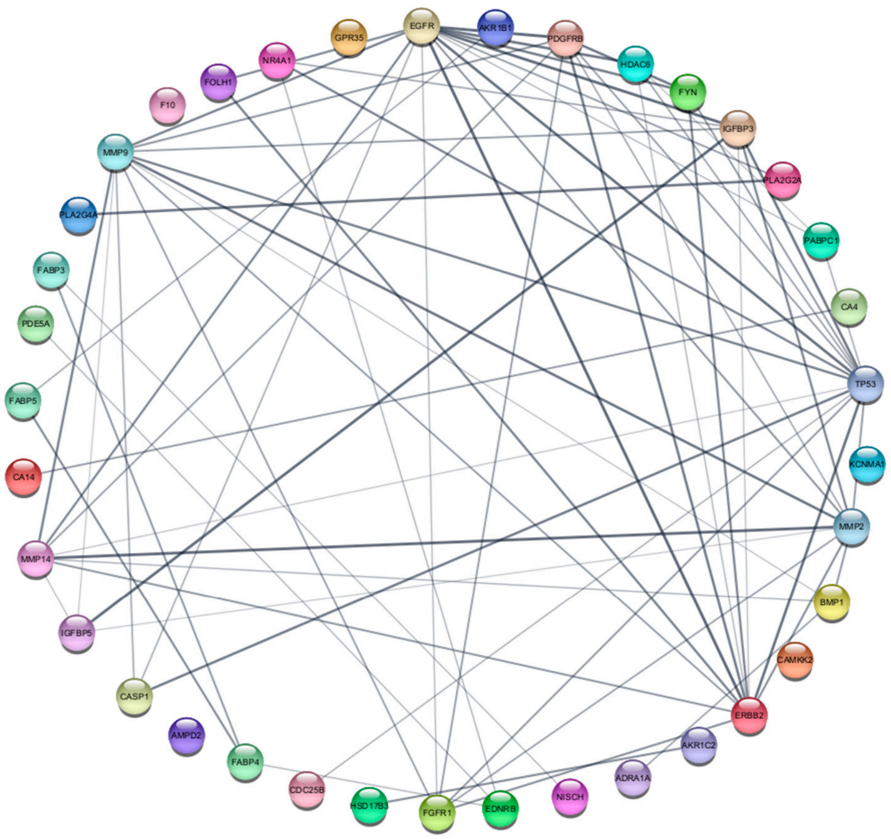

Figure S1. PPI Network from 37 intersected targets for PCa with an interaction score of 0.04

GO AND PATHWAY ENRICHMENT ANALYSIS

Table S3a. GO enrichment analysis on biological process

Gene Set	Description	P Value	FDR
GO:0001934	positive regulation of protein phosphorylation	8.88E-10	8.19E-07
GO:0008283	cell proliferation	6.45E-10	8.19E-07
GO:0009967	positive regulation of signal transduction	9.76E-11	8.19E-07
GO:0010562	positive regulation of phosphorus metabolic process	3.85E-10	8.19E-07
GO:0010647	positive regulation of cell communication	5.31E-10	8.19E-07
GO:0023056	positive regulation of signaling	5.63E-10	8.19E-07
GO:0032270	positive regulation of cellular protein metabolic process	7.23E-10	8.19E-07
GO:0042127	regulation of cell proliferation	9.01E-10	8.19E-07
GO:0045937	positive regulation of phosphate metabolic process	3.85E-10	8.19E-07
GO:1902533	positive regulation of intracellular signal transduction	2.05E-10	8.19E-07
GO:0042327	positive regulation of phosphorylation	1.67E-09	1.33E-06
GO:0051897	positive regulation of protein kinase B signaling	1.76E-09	1.33E-06
GO:0051247	positive regulation of protein metabolic process	1.94E-09	1.36E-06
GO:0033002	muscle cell proliferation	5.11E-09	3.32E-06
GO:0001932	regulation of protein phosphorylation	1.18E-08	6.40E-06
GO:0031401	positive regulation of protein modification process	1.23E-08	6.40E-06
GO:0048659	smooth muscle cell proliferation	1.27E-08	6.40E-06
GO:0048660	regulation of smooth muscle cell proliferation	1.14E-08	6.40E-06
GO:0051896	regulation of protein kinase B signaling	1.40E-08	6.71E-06
GO:0019220	regulation of phosphate metabolic process	2.57E-08	1.08E-05
GO:0048661	positive regulation of smooth muscle cell proliferation	2.43E-08	1.08E-05
GO:0051174	regulation of phosphorus metabolic process	2.62E-08	1.08E-05
GO:0042325	regulation of phosphorylation	3.55E-08	1.34E-05
GO:0043491	protein kinase B signaling	3.41E-08	1.34E-05
GO:0046486	glycerolipid metabolic process	3.83E-08	1.39E-05
GO:1901700	response to oxygen-containing compound	5.95E-08	2.08E-05
GO:0006650	glycerophospholipid metabolic process	8.16E-08	2.64E-05
GO:0009719	response to endogenous stimulus	8.43E-08	2.64E-05
GO:1902531	regulation of intracellular signal transduction	8.00E-08	2.64E-05
GO:1901701	cellular response to oxygen-containing compound	2.56E-07	7.76E-05
GO:0008284	positive regulation of cell proliferation	2.95E-07	8.66E-05
GO:0031399	regulation of protein modification process	3.61E-07	1.02E-04
GO:0009725	response to hormone	6.18E-07	1.70E-04
GO:0006644	phospholipid metabolic process	6.45E-07	1.72E-04
GO:0006468	protein phosphorylation	7.06E-07	1.79E-04

Table S3b. GO enrichment analysis on biological process

Gene Set	Description	P Value	FDR
GO:0046488	phosphatidylinositol metabolic process	7.08E-07	1.79E-04
GO:0050673	epithelial cell proliferation	1.25E-06	3.08E-04
GO:0018108	peptidyl-tyrosine phosphorylation	1.65E-06	3.89E-04
GO:2000145	regulation of cell motility	1.67E-06	3.89E-04
GO:0018212	peptidyl-tyrosine modification	1.75E-06	3.98E-04
GO:0051674	localization of cell	2.09E-06	4.51E-04
GO:0010863	positive regulation of phospholipase C activity	2.17E-06	4.58E-04
GO:0046854	phosphatidylinositol phosphorylation	4.60E-06	8.05E-04
GO:0006629	lipid metabolic process	4.94E-06	8.48E-04
GO:0051240	positive regulation of multicellular organismal process	6.05E-06	1.02E-03
GO:0016049	cell growth	7.43E-06	1.14E-03
GO:0030335	positive regulation of cell migration	7.32E-06	1.14E-03
GO:0015909	long-chain fatty acid transport	1.26E-05	1.57E-03
GO:0070374	positive regulation of ERK1 and ERK2 cascade	1.19E-05	1.57E-03
GO:0045860	positive regulation of protein kinase activity	1.34E-05	1.65E-03
GO:0048878	chemical homeostasis	2.01E-05	2.15E-03
GO:0006811	ion transport	2.32E-05	2.37E-03
GO:0019637	organophosphate metabolic process	2.55E-05	2.55E-03
GO:0030324	lung development	3.29E-05	3.03E-03
GO:2000377	regulation of reactive oxygen species metabolic process	3.49E-05	3.17E-03
GO:1905475	regulation of protein localization to membrane	4.23E-05	3.68E-03
GO:0019433	triglyceride catabolic process	4.78E-05	4.06E-03
GO:1904707	positive regulation of vascular smooth muscle cell proliferation	4.78E-05	4.06E-03
GO:0042981	regulation of apoptotic process	6.59E-05	5.16E-03
GO:1903997	positive regulation of non-membrane spanning protein tyrosine kinase activity	1.00E-04	7.28E-03
GO:0030574	collagen catabolic process	1.01E-04	7.31E-03
GO:0003014	renal system process	1.11E-04	7.85E-03
GO:0010822	positive regulation of mitochondrion organization	1.15E-04	8.06E-03
GO:0010727	negative regulation of hydrogen peroxide metabolic process	1.33E-04	8.83E-03
GO:0044598	doxorubicin metabolic process	1.33E-04	8.83E-03
GO:0022414	reproductive process	1.59E-04	1.03E-02
GO:0010243	response to organonitrogen compound	1.72E-04	1.07E-02
GO:0019318	hexose metabolic process	1.71E-04	1.07E-02
GO:1903800	positive regulation of production of miRNAs involved in gene silencing by miRNA	1.71E-04	1.07E-02
GO:0046474	glycerophospholipid biosynthetic process	1.85E-04	1.12E-02

Table S3c. GO enrichment analysis on biological process

Gene Set	Description	P Value	FDR
GO:0009968	negative regulation of signal transduction	2.11E-04	1.26E-02
GO:0043406	positive regulation of MAP kinase activity	2.49E-04	1.42E-02
GO:0014910	regulation of smooth muscle cell migration	3.01E-04	1.66E-02
GO:0043568	positive regulation of insulin-like growth factor receptor signaling pathway	3.68E-04	1.93E-02
GO:0042310	vasoconstriction	5.19E-04	2.56E-02
GO:0036149	phosphatidylinositol acyl-chain remodeling	6.39E-04	2.93E-02
GO:0050679	positive regulation of epithelial cell proliferation	7.00E-04	3.12E-02
GO:0048145	regulation of fibroblast proliferation	8.19E-04	3.40E-02
GO:0042692	muscle cell differentiation	8.52E-04	3.46E-02
GO:0036120	cellular response to platelet-derived growth factor stimulus	8.89E-04	3.53E-02
GO:0071229	cellular response to acid chemical	1.03E-03	3.95E-02
GO:0032101	regulation of response to external stimulus	1.35E-03	4.83E-02
GO:0046474	glycerophospholipid biosynthetic process	1.85E-04	1.12E-02

Table S4. GO enrichment analysis on cellular component

Gene Set	Description	P Value	FDR
GO:0045121	membrane raft	1.79E-05	6.26E-03
GO:0098857	membrane microdomain	1.82E-05	6.26E-03
GO:0031226	intrinsic component of plasma membrane	1.94E-05	6.26E-03
GO:0098589	membrane region	2.25E-05	6.26E-03
GO:0016942	insulin-like growth factor binding protein complex	3.19E-05	6.26E-03
GO:0036454	growth factor complex	3.19E-05	6.26E-03
GO:0048471	perinuclear region of cytoplasm	1.87E-04	3.14E-02
GO:0016324	apical plasma membrane	2.27E-04	3.34E-02
GO:0005887	integral component of plasma membrane	3.67E-04	4.62E-02
GO:0005901	caveola	3.94E-04	4.62E-02

Table S5a. GO enrichment analysis on molecular function

Gene Set	Description	P Value	FDR
GO:0046934	phosphatidylinositol-4,5-bisphosphate 3-kinase activity	4.77E-07	3.67E-04
GO:0019838	growth factor binding	5.44E-07	3.67E-04
GO:0052813	phosphatidylinositol bisphosphate kinase activity	5.87E-07	3.67E-04
GO:0035004	phosphatidylinositol 3-kinase activity	9.81E-07	4.60E-04
GO:0004713	protein tyrosine kinase activity	2.41E-06	9.04E-04
GO:0004714	transmembrane receptor protein tyrosine kinase activity	1.19E-05	3.71E-03
GO:0033293	monocarboxylic acid binding	1.90E-05	5.09E-03

Table S5b. GO enrichment analysis on molecular function

Gene Set	Description	P Value	FDR
GO:0019199	transmembrane receptor protein kinase activity	3.03E-05	7.10E-03
GO:0004252	serine-type endopeptidase activity	4.82E-05	8.90E-03
GO:0008270	zinc ion binding	4.87E-05	8.90E-03
GO:0008237	metallopeptidase activity	5.21E-05	8.90E-03
GO:0005504	fatty acid binding	5.75E-05	8.99E-03
GO:0005102	signaling receptor binding	7.57E-05	1.09E-02
GO:0008236	serine-type peptidase activity	8.29E-05	1.11E-02
GO:0017171	serine hydrolase activity	9.08E-05	1.14E-02
GO:0004222	metalloendopeptidase activity	1.00E-04	1.18E-02
GO:0031995	insulin-like growth factor II binding	1.33E-04	1.47E-02
GO:0005088	Ras guanyl-nucleotide exchange factor activity	2.35E-04	2.45E-02
GO:0005324	long-chain fatty acid transporter activity	3.12E-04	2.88E-02
GO:0031994	insulin-like growth factor I binding	3.12E-04	2.88E-02
GO:0070011	peptidase activity, acting on L-amino acid peptides	3.36E-04	2.88E-02
GO:0004175	endopeptidase activity	3.62E-04	2.88E-02
GO:0004032	alditol:NADP+ 1-oxidoreductase activity	3.68E-04	2.88E-02
GO:0036041	long-chain fatty acid binding	3.68E-04	2.88E-02
GO:0046914	transition metal ion binding	3.99E-04	3.00E-02
GO:0008233	peptidase activity	4.24E-04	3.06E-02
GO:0004089	carbonate dehydratase activity	4.94E-04	3.31E-02
GO:0047498	calcium-dependent phospholipase A2 activity	4.94E-04	3.31E-02
GO:0005085	guanyl-nucleotide exchange factor activity	7.54E-04	4.88E-02

Table S6. KEGG pathway enrichment analysis

Gene Set	Description	Ratio	P Value	FDR
hsa05219	Bladder cancer	26.08	1.1E-06	3.57E-04
hsa05215	Prostate cancer	13.228	4.8E-06	7.82E-04
hsa05230	Central carbon metabolism in cancer	16.45	1.1E-05	1.21E-03
hsa04010	MAPK signaling pathway	5.7994	4.8E-05	3.79E-03
hsa05206	MicroRNAs in cancer	8.5541	5.8E-05	3.79E-03
hsa01522	Endocrine resistance	10.911	8.2E-05	4.45E-03
hsa05205	Proteoglycans in cancer	6.4804	0.00027	1.19E-02
hsa04520	Adherens junction	11.881	0.00033	1.19E-02
hsa05218	Melanoma	11.881	0.00033	1.19E-02
hsa04912	GnRH signaling pathway	9.198	0.00087	2.83E-02
hsa04020	Calcium signaling pathway	5.843	0.00147	4.36E-02

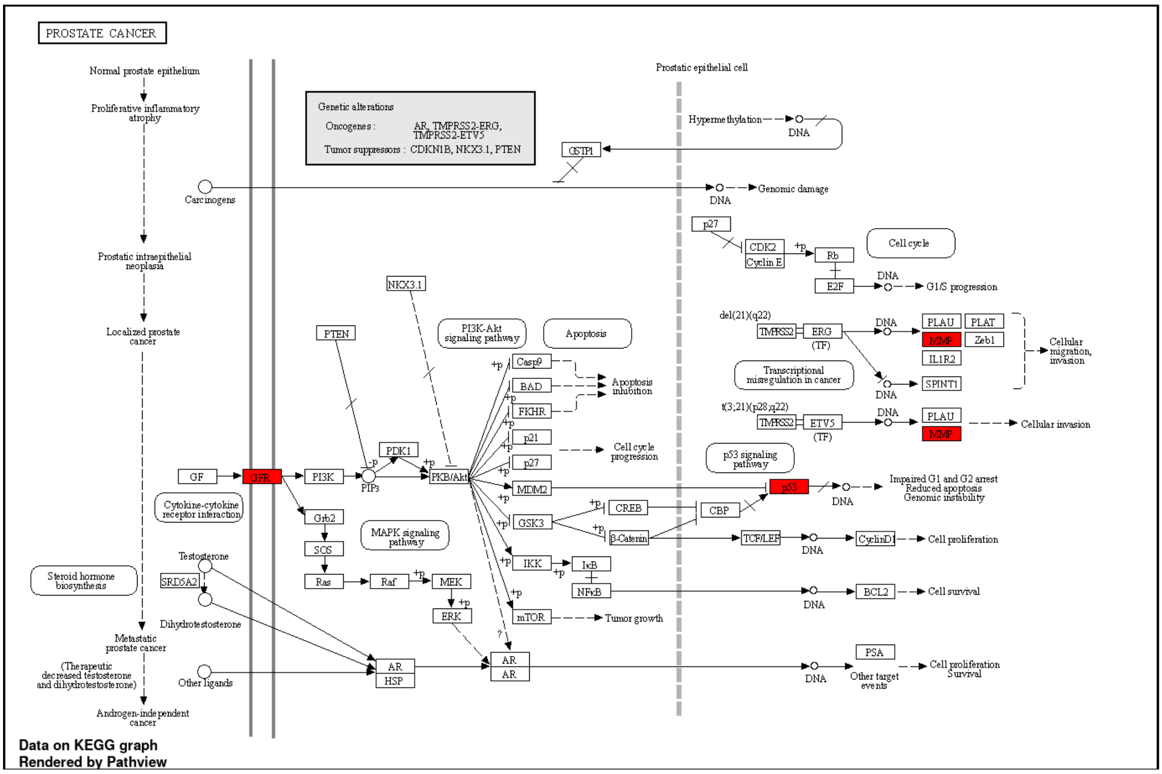

Figure S2. Prostate cancer pathway from KEGG pathway

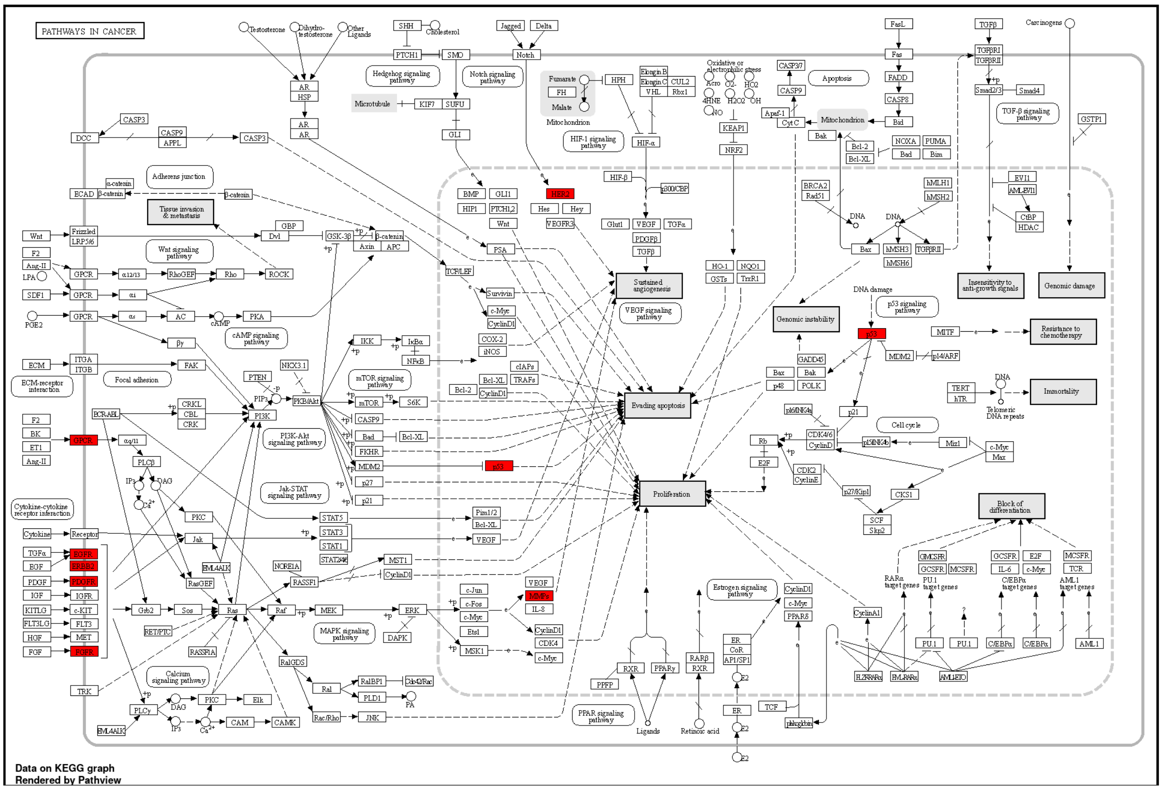

Figure S3. Pathways in cancer from KEGG pathway

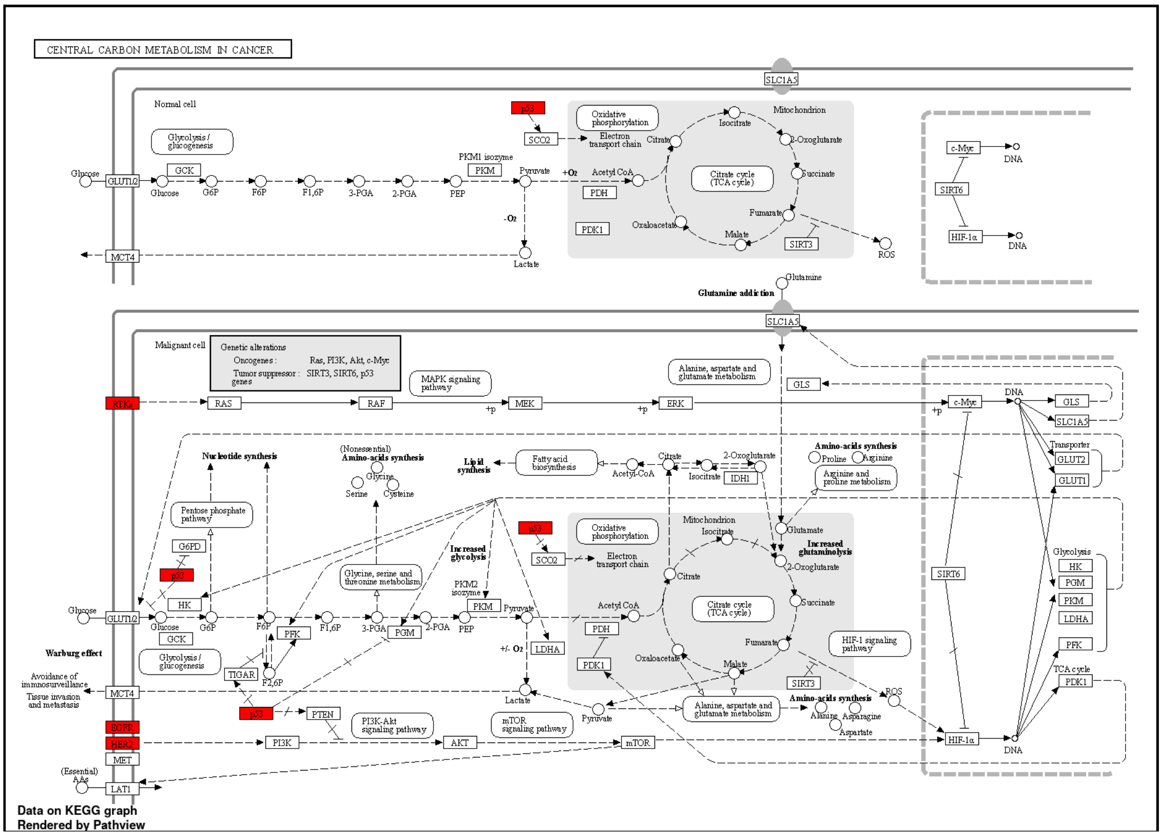

Figure S4. Central carbon metabolism in cancer from KEGG pathway

Table S7. Reactome pathway enrichment analysis

Gene Set	Description	Ratio	P Value	FDR
R-HSA-2219528	PI3K/AKT Signaling in Cancer	18.65	6.75E-07	1.17E-03
R-HSA-2219530	Constitutive Signaling by Aberrant PI3K in Cancer	21.23	3.32E-06	2.87E-03
R-HSA-6811558	PI5P, PP2A and IER3 Regulate PI3K/AKT Signaling	15.23	1.70E-05	8.18E-03
R-HSA-1257604	PIP3 activates AKT signaling	8.12	1.89E-05	8.18E-03
R-HSA-199418	Negative regulation of the PI3K/AKT network	14.22	2.38E-05	8.21E-03
R-HSA-9006925	Intracellular signaling by second messengers	7.20	4.08E-05	1.18E-02
R-HSA-163560	Triglyceride catabolism	37.69	6.45E-05	1.59E-02
R-HSA-1592389	Activation of Matrix Metalloproteinases	27.41	1.70E-04	3.68E-02
R-HSA-5663202	Diseases of signal transduction	5.58	2.02E-04	3.88E-02
R-HSA-8979227	Triglyceride metabolism	24.45	2.40E-04	4.15E-02

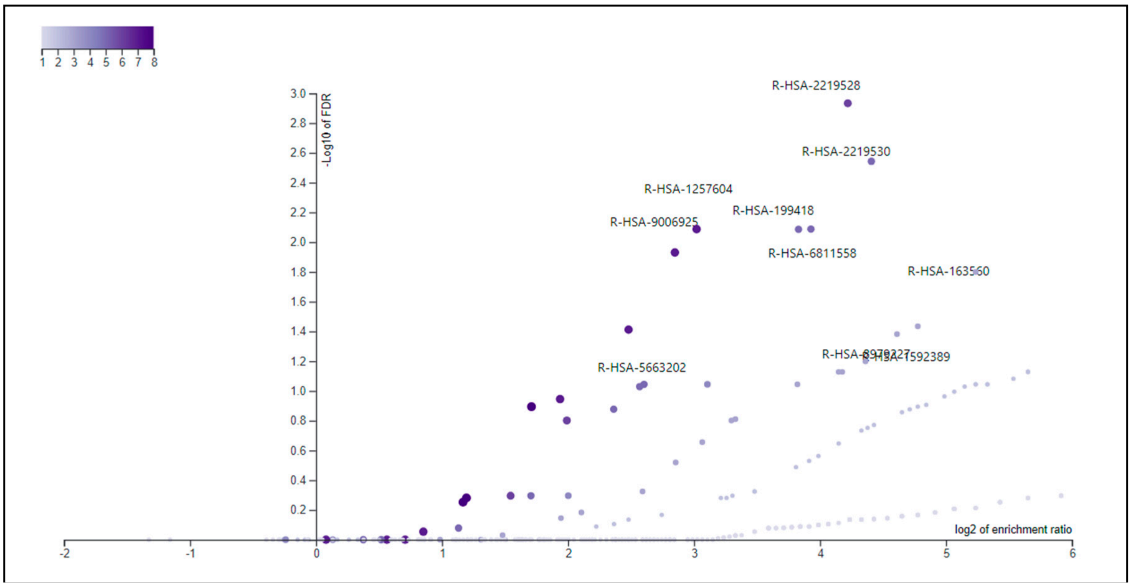

Figure S5. Volcano plot of enriched results for Reactome pathway enrichment analysis

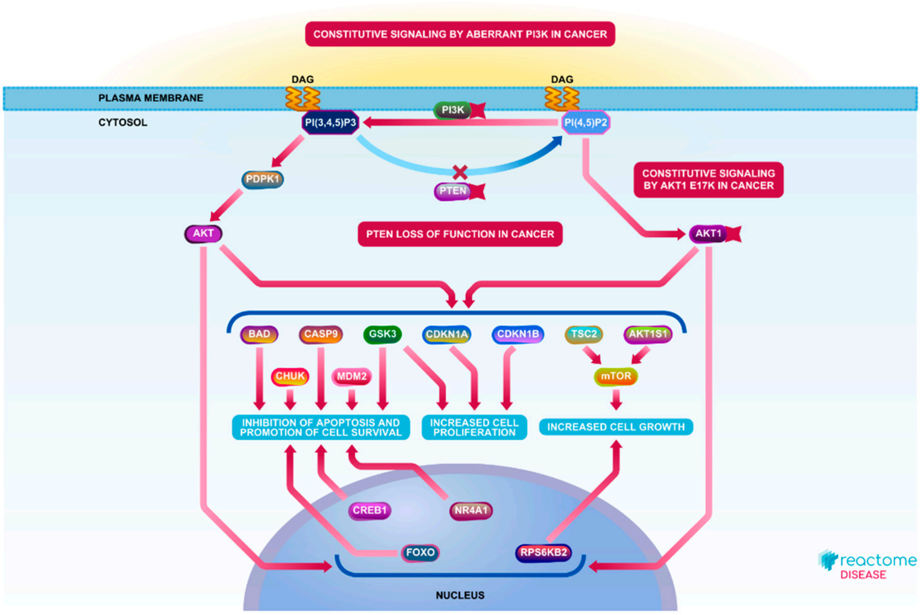

Figure S6. PI3K/AKT signaling in cancer from Reactome Pathway Database

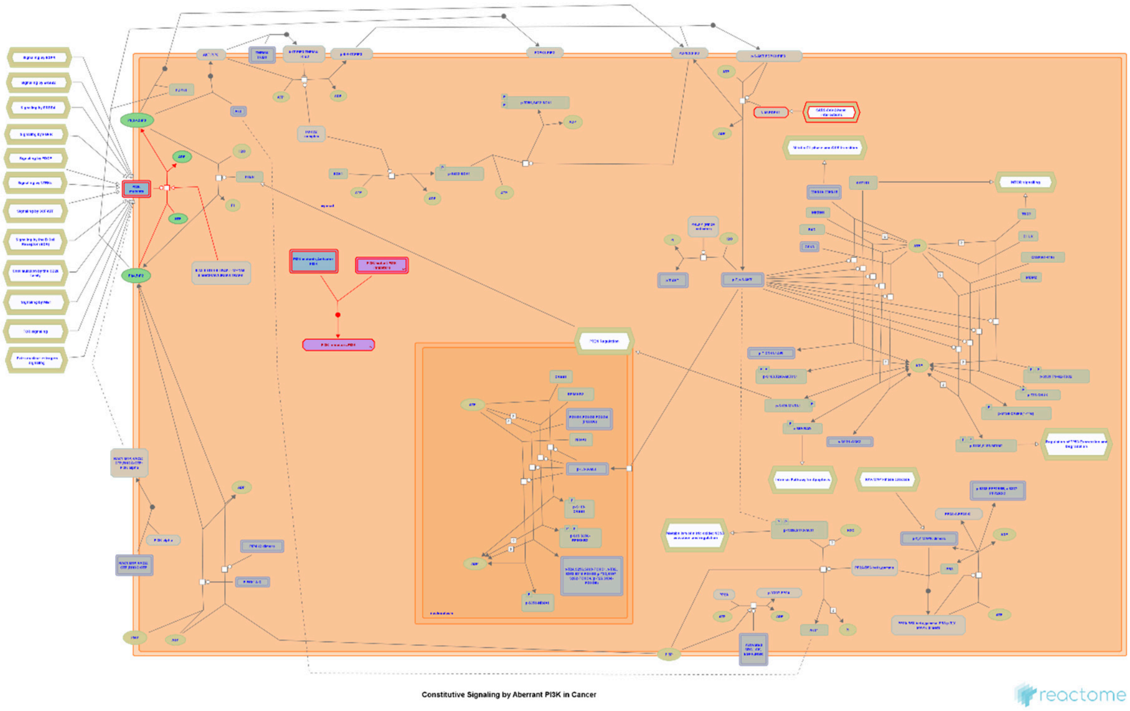

Figure S7. Constitutive signaling by aberrant PI3K in cancer from Reactome Pathway Database

BINDING SITE COORDINATES FOR MOLECULAR DOCKING

Table S8. Receptor Spatial Coordinate

Receptor	x Coordinate	y Coordinate	z Coordinate
EGFR	21.24	0.57	52.33
ERBB2	35.26	44.22	-11.02
FGFR1	-47.73	-4.09	34.04
HDAC6	-1.13	8.87	5.8
MMP2	5.67	18.85	22.41
MMP9	65.78	30.9	117.99
TP53	124.76	105.37	-42.96
PDGFRB	11.07	-37.68	55.74
IGFBP3	132.21	142.19	147.85

2D RECEPTOR-LIGAND INTERACTIONS

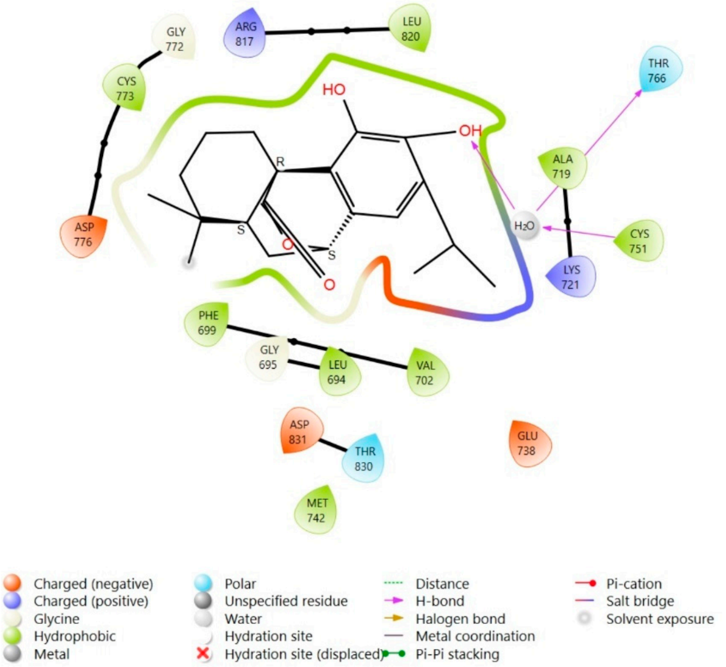

Figure S8a. 2D Receptor-Ligand Interactions of EGFR-COH complex from Maestro

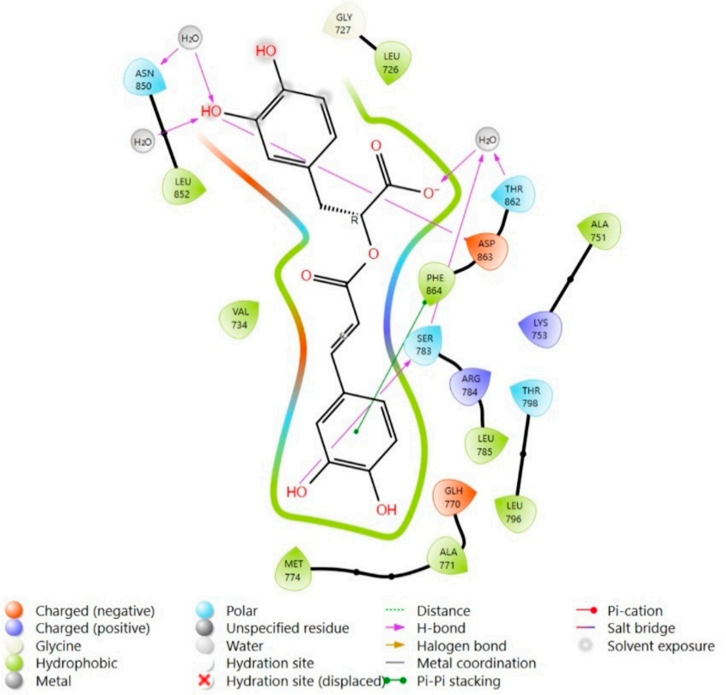

Figure S8b. 2D Receptor-Ligand Interactions of ERBB2-RA complex from Maestro

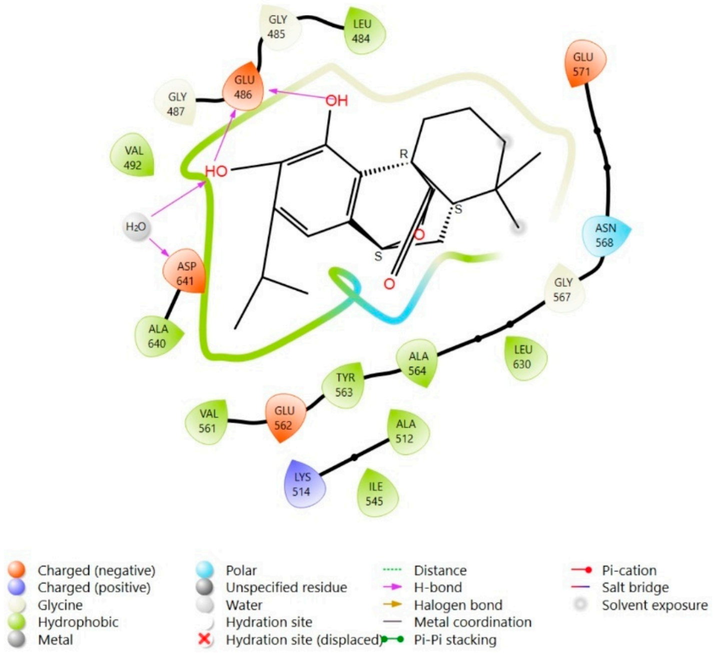

Figure S8c. 2D Receptor-Ligand Interactions of FGFR1-COH complex with from Maestro

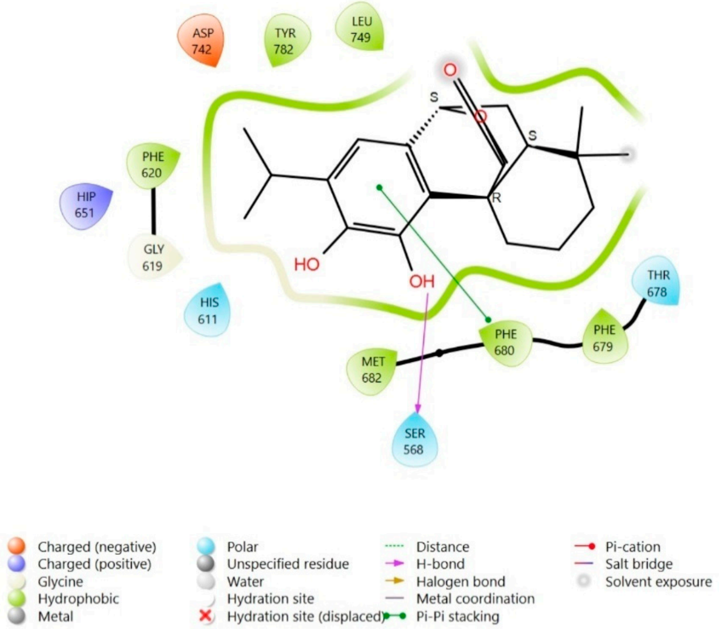

Figure S8d. 2D Receptor-Ligand Interactions of HDAC6-COH complex from Maestro

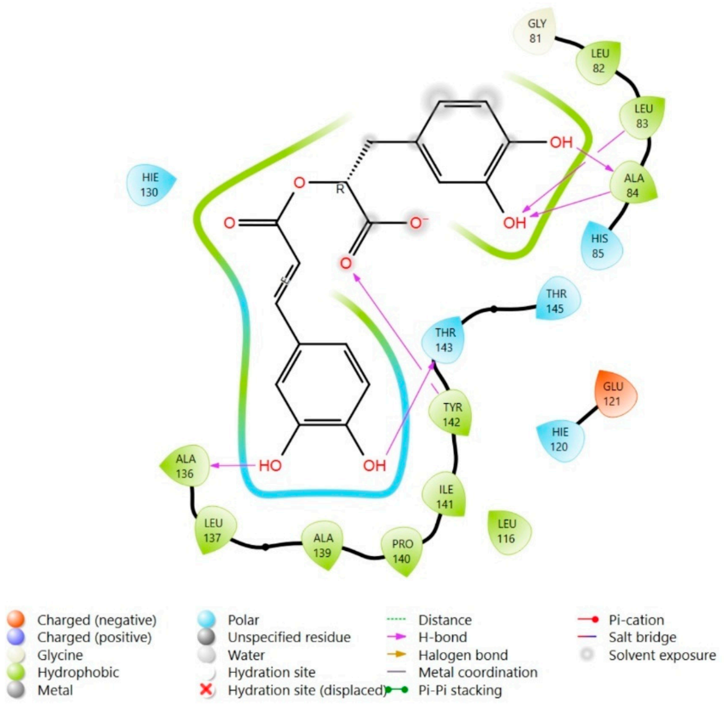

Figure S8e. 2D Receptor-Ligand Interactions of MMP2-RA complex from Maestro

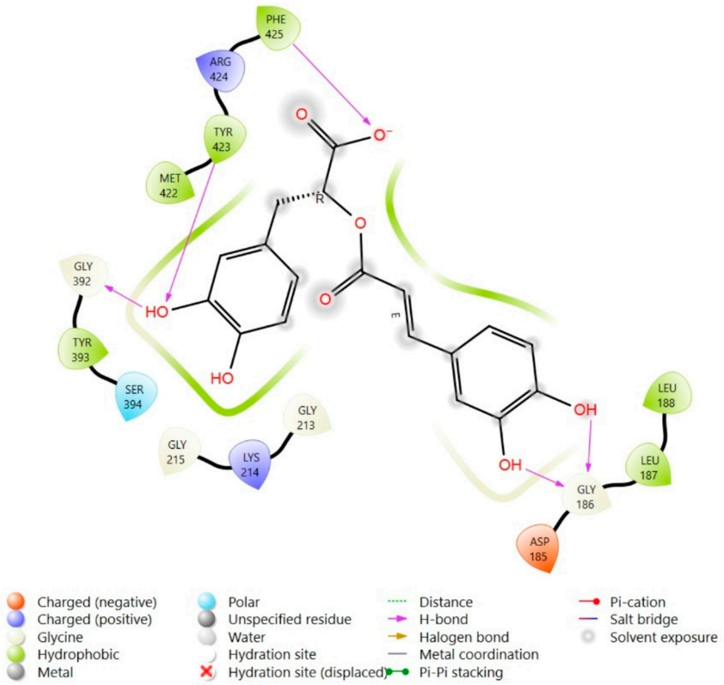

Figure S8f. 2D Receptor-Ligand Interactions of MMP9-RA complex from Maestro

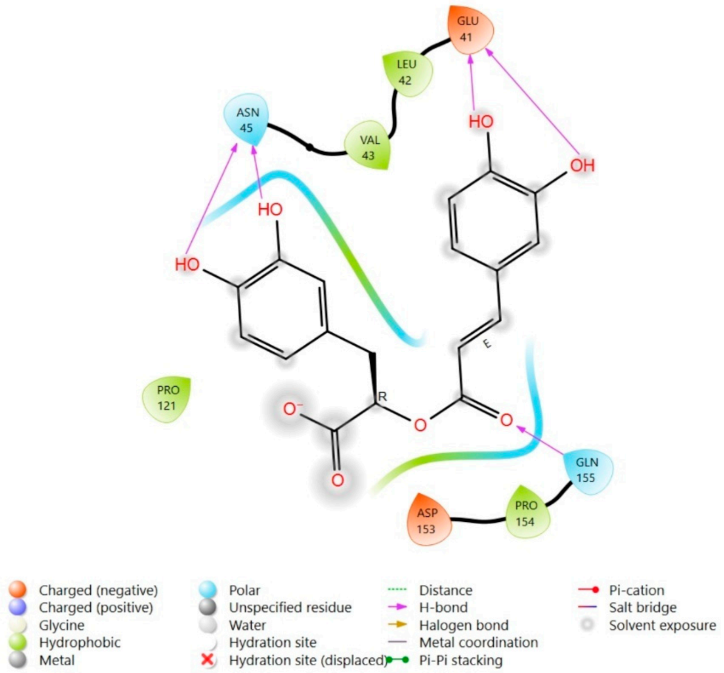

Figure S8g. 2D Receptor-Ligand Interactions of PDGFRB-RA complex from Maestro

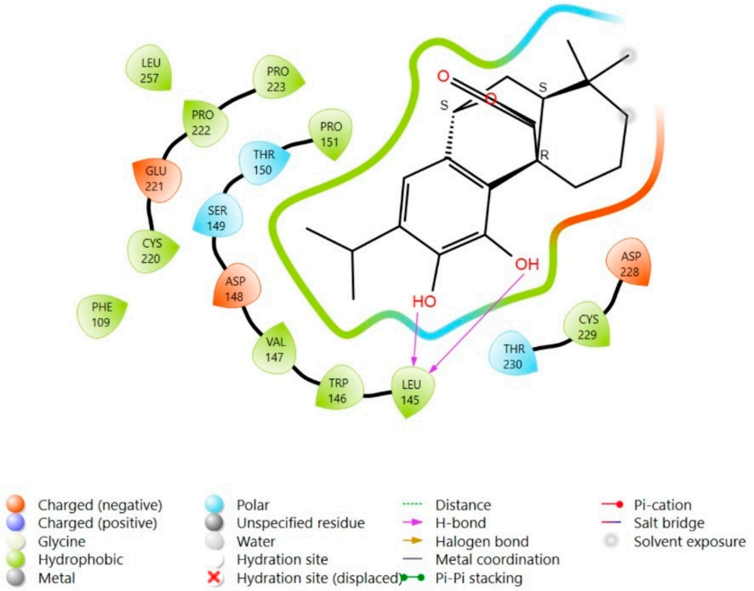

Figure S8h. 2D Receptor-Ligand Interactions of TP53-COH complex from Maestro

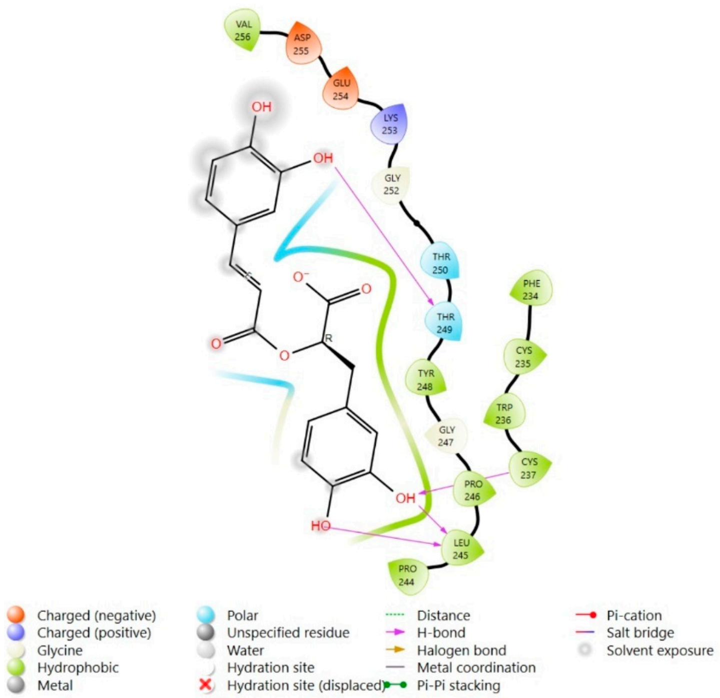

Figure S8i. 2D Receptor-Ligand Interactions of IGFBP3-RA complex from Maestro

MOLECULAR DYNAMICS SIMULATION REPORTS

a. EGFR-COH complex

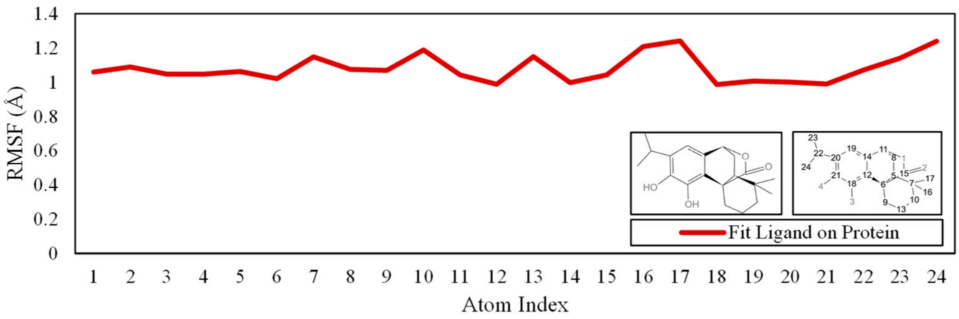

Figure S9. Ligand RMSF analysis for EGFR-COH complex over 100 ns

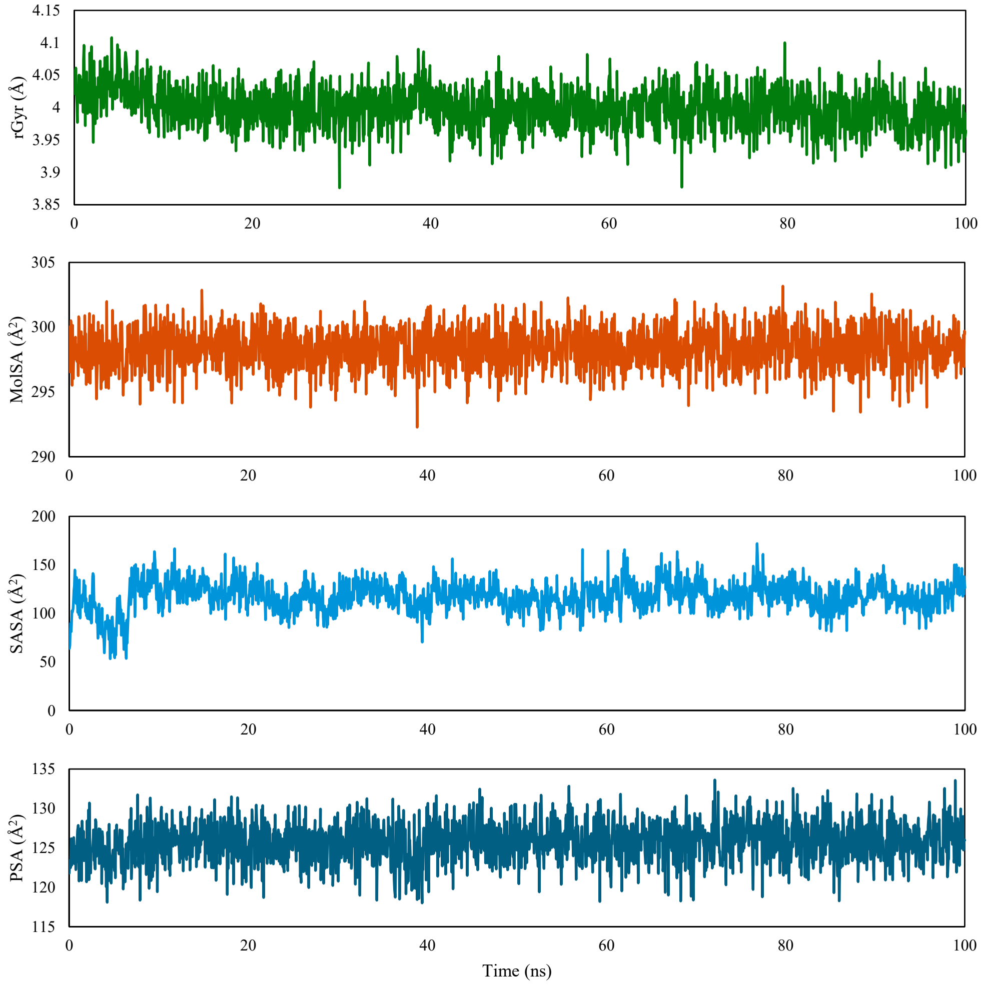

Figure S10. Ligand properties analysis of EGFR-COH complex over 100 ns

b. ERBB2-RA complex

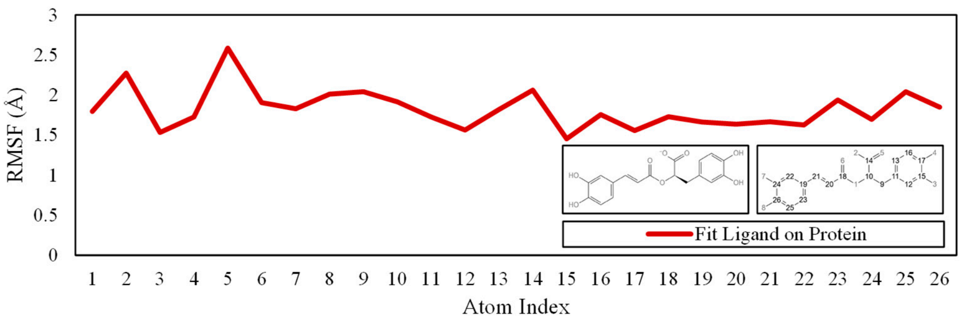

Figure S11. Ligand RMSF analysis for ERBB2-RA complex over 100 ns

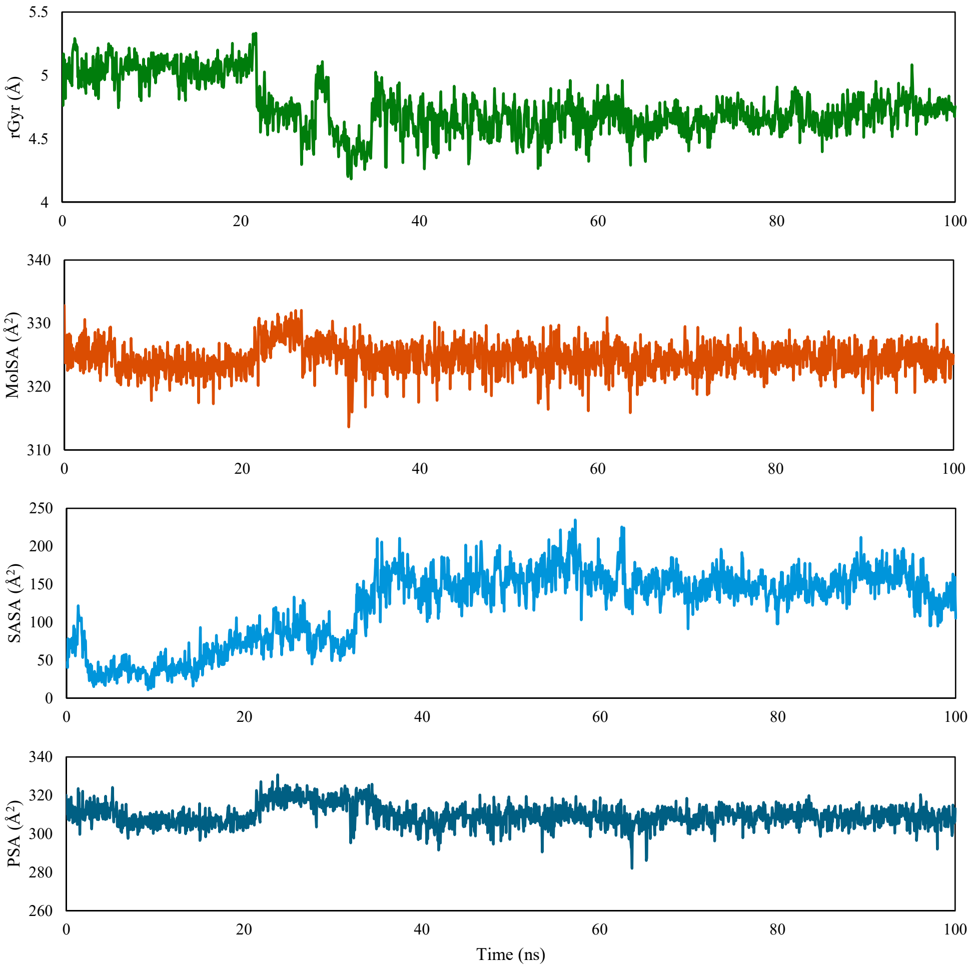

Figure S12. Ligand properties analysis of ERBB2-RA complex over 100 ns

c. TP53-COH complex

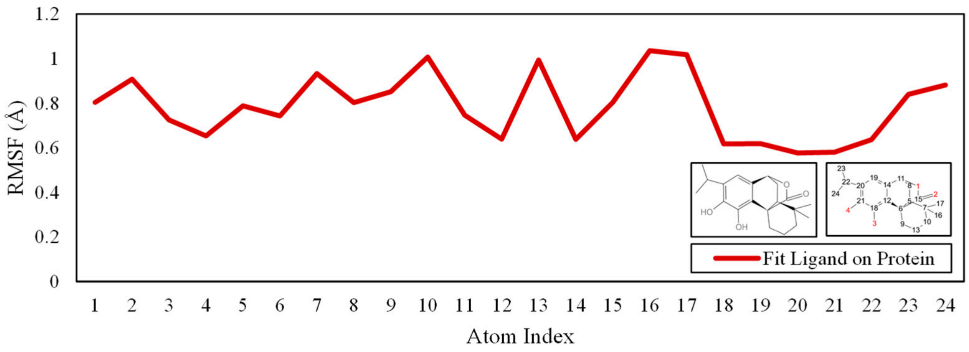

Figure S13. Ligand RMSF analysis for TP53-COH complex over 100 ns

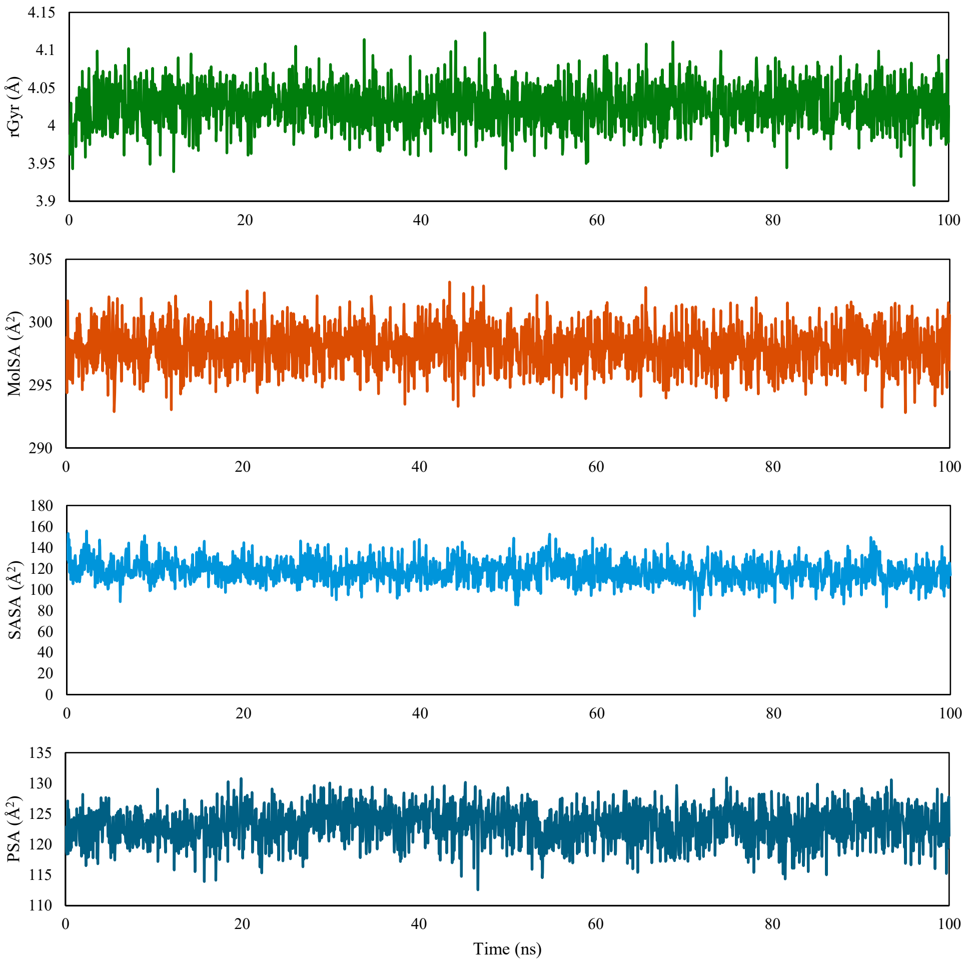

Figure S14. Ligand properties analysis of TP53-COH complex over 100 ns
